# Stiffness‐Activated Stellate Cells Drive Pancreatic Cancer Liver Colonization via GMFG‐TNS4 Signaling

**DOI:** 10.1002/advs.76173

**Published:** 2026-06-18

**Authors:** Biwen Zhu, Jian Wan, Xue Zhang, Jiashuai Yan, Xi Chen, Xiaoqi Guan, Qianqian Ren, Tao Yang, Di Wu, Qingsong Guo, Yibing Guo, Yuhua Lu

**Affiliations:** ^1^ Department of Hepatobiliary and Pancreatic Surgery Affiliated Hospital and Medical School of Nantong University Nantong China; ^2^ Research Center of Clinical Medicine Affiliated Hospital and Medical School of Nantong University Nantong China; ^3^ Co‐innovation Center of Neuro‐regeneration Key Laboratory of Neuro‐regeneration of Jiangsu and Ministry of Education Nantong China; ^4^ Department of Otorhinolaryngology Head and Neck surgery Affiliated Hospital of Nantong University Nantong China

**Keywords:** liver metastasis, matrix stiffness, pancreatic ductal adenocarcinoma (PDAC), Piezo1‐GMFG‐TNS4 axis

## Abstract

Liver metastasis remains the primary cause of death in pancreatic cancer. Collagen deposition by activated hepatic stellate cells (HSCs) generates a stiff fibrotic niche that favors metastatic colonization, yet the underlying mechanisms remain incompletely understood. Using stiffness‐tunable hydrogels, it is shown that elevated substrate stiffness activates HSCs and establishes a self‐reinforcing loop of matrix stiffening. Mechanistically, stiffness triggers Piezo1‐mediated Ca^2^^+^ influx, induces endoplasmic reticulum stress (ERS), and activates the IRE1α‐XBP1 pathway to upregulate glia maturation factor gamma (GMFG) transcription and secretion. GMFG is transported into pancreatic cancer cells where it binds to intracellular tensin‐4 (TNS4), promoting FAK/AKT phosphorylation and coordinating two programs critical for metastatic outgrowth: enhanced cell‐ECM adhesion and increased de novo fatty acid synthesis. In mice with graded liver stiffness, pharmacological inhibition of mechanosensitive cation channels reduces metastatic burden and dampens GMFG‐associated epithelial and lipogenic features, while targeting the GMFG‐TNS4 axis suppresses early hepatic micrometastatic seeding and long‐term liver metastasis burden. Together, these findings define a mechano‐ER stress‐paracrine cascade linking fibrotic stiffness to pro‐colonization signaling, highlighting the Piezo1‐GMFG‐TNS4 pathway as a therapeutic vulnerability in PDAC liver metastasis.

## Graphical abstract

Fibrotic liver stiffness activates hepatic stellate cells through Piezo1‐dependent calcium influx and ER stress, promoting EV‐associated GMFG release. Delivered GMFG engages TNS4 in pancreatic cancer cells, triggering FAK/AKT signaling, adhesion, and fatty acid synthesis. This mechano‐paracrine cascade establishes a targetable niche program that supports liver colonization and metastatic outgrowth.

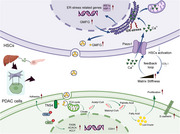

## 1 Introduction

Pancreatic ductal adenocarcinoma (PDAC) is among the most lethal malignancies, and liver metastasis accounts for the majority of PDAC‐related deaths [1, 2, 3]. The “seed and soil” framework emphasizes that metastatic success depends not only on disseminated tumor cells but also on organ‐specific microenvironments that enable seeding, survival, and outgrowth [4, 5]. In the liver, pancreatic cancer cells encounter a stromal niche shaped by hepatic stellate cells (HSCs), resident immune cells, and dynamic extracellular matrix (ECM) remodeling [6]. Defining the drivers that render the liver permissive for PDAC colonization is therefore critical for developing effective anti‐metastatic strategies.

A hallmark of pro‐metastatic liver niches is fibrotic remodeling, in which activated HSCs acquire a myofibroblast‐like state and deposit collagen‐rich ECM, increasing tissue stiffness [7, 8, 9, 10]. Mechanical cues have emerged as instructive signals in cancer progression, and mechanotransduction hubs such as YAP/TAZ and integrins have been implicated in stiffness‐associated malignancy [11, 12, 13, 14, 15, 16]. Emerging clinical and translational evidence suggests that the hepatic mechanical background is clinically relevant to PDAC liver metastasis. In patients with pancreatic cancer liver metastasis, higher liver lesion stiffness measured by two‐dimensional shear wave elastography has been associated with worse progression‐free survival [17]. In parallel, liver stiffening can arise early during metabolic dysfunction‐associated steatotic liver disease (MASLD), even before overt fibrosis becomes histologically apparent, and MASLD has recently been linked to increased PDAC risk, a higher frequency of hepatic metastasis, and faster liver‐specific metastatic progression [18, 19]. These observations support the concept that a chronically stiffened liver microenvironment may favor PDAC liver colonization. However, it remains unclear how fibrotic stiffness is sensed by HSCs and translated into specific biochemical outputs that instruct PDAC colonization.

Metastatic outgrowth in the liver requires tumor cells to rapidly establish physical anchorage and adapt their biosynthetic metabolism to the hepatic environment [20, 21, 22, 23, 24, 25, 26]. Notably, accumulating evidence indicates that metastatic lesions often reacquire epithelial traits during colonization (a MET‐like program), and E‐cadherin has been shown to facilitate hepatic outgrowth in PDAC models [27, 28, 29, 30, 31, 32, 33]. In parallel, liver metastases undergo extensive metabolic rewiring, including altered lipid flux that supports rapid proliferation and survival [34, 35]. However, whether and how stiffness‐educated stromal cells coordinate adhesion‐related programs with metabolic adaptation in PDAC cells during early colonization remains largely unknown.

Here, using stiffness‐tunable GelMA hydrogels and stiffness‐graded liver metastasis models, we show that elevated stiffness activates HSCs through Piezo1‐dependent Ca^2^^+^ influx and establishes a collagen‐driven feed‐forward stiffening loop. Stiffness‐induced ERS activates the IRE1α‐XBP1 axis to promote GMFG transcription and secretion from activated HSCs. GMFG then engages intracellular TNS4 in PDAC cells to trigger FAK‐AKT signaling, thereby enhancing cell‐ECM adhesion and fatty acid synthesis to support hepatic colonization and metastatic outgrowth. These findings reveal a mechano‐to‐paracrine signaling cascade linking fibrotic stiffness to pro‐colonization programs in PDAC liver metastasis.

## 2 Results

### 2.1 Stiff‐Liver Background Promotes Pancreatic Cancer Liver Metastasis

**FIGURE 1:**
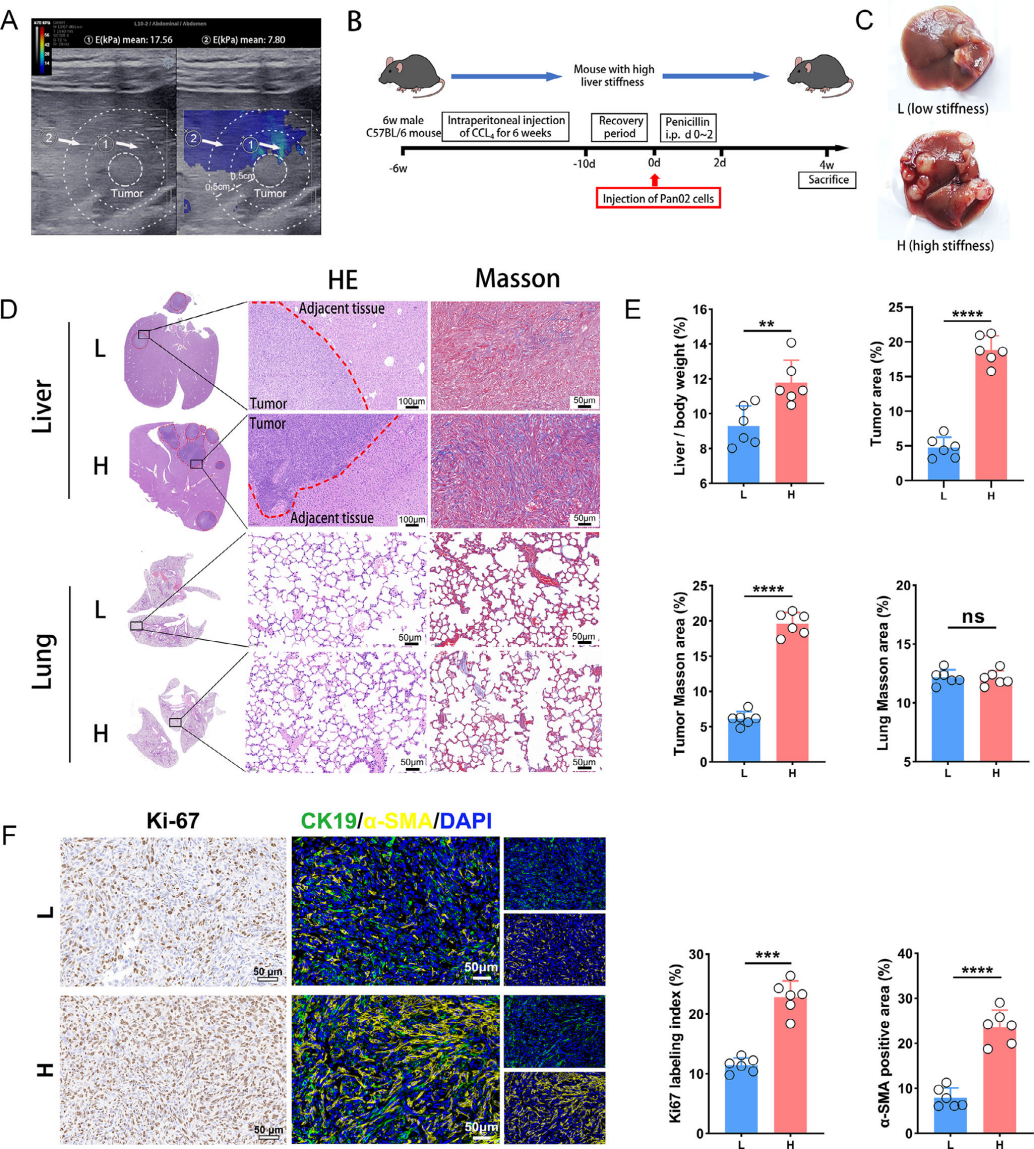
A spatial liver‐stiffness gradient associates with pancreatic cancer liver metastasis and a fibrotic liver microenvironment promotes metastatic outgrowth in vivo. (A) Representative ultrasound shear‐wave elastography (SWE) maps of human pancreatic cancer liver metastases. (B) Schematic of the carbon tetrachloride (CCl_4_)–induced high‐stiffness liver model and subsequent establishment of liver metastasis. (C) Representative images of livers from low‐stiffness (L) and high‐stiffness (H) groups at endpoint. (D) Representative H&E and Masson's trichrome staining of liver metastatic lesions and lung sections. (E) Quantification of liver‐to‐body weight ratio, tumor area per liver section, Masson‐positive area within liver metastatic lesions, and Masson‐positive area in lungs (*n* = 6). (F) Representative Ki67 immunohistochemistry (IHC), cytokeratin 19 (CK19), and α‐smooth muscle actin (α‐SMA) immunofluorescence staining of liver metastatic lesions with corresponding quantification (*n* = 6). Scale bars are indicated in the images. All data are presented as mean ± SD. Statistical analysis was performed using two‐tailed Student's t‐test (two groups) or one‐way ANOVA. **p < 0.01, ***p < 0.001, ****p < 0.0001.

We first asked whether liver mechanics are altered in patients with pancreatic cancer liver metastasis. Ultrasound shear‐wave elastography (SWE) revealed a pronounced peritumoral stiffness gradient surrounding metastatic lesions, with the highest stiffness detected adjacent to the tumor border and progressively lower values with increasing distance (Figure 1A). This spatial pattern was further supported by nanoscale indentation measurements, confirming that metastatic foci reside within a locally stiffened hepatic microenvironment (Figure S1A,B). These clinical observations prompted us to test whether a stiff liver “soil” is sufficient to facilitate metastatic outgrowth.

To test whether a stiff hepatic microenvironment promotes metastatic outgrowth, we generated a stiffness‐graded liver model using carbon tetrachloride (CCl_4_) followed by a recovery period before tumor inoculation (Figure 1B and Figure S1E). To minimize confounding from CCl_4_‐induced liver injury and inflammation, we assessed liver status before tumor challenge. Serum alanine aminotransferase (ALT) and aspartate aminotransferase (AST) levels did not differ between groups, and Ly6G^+^ and F4/80^+^ immune signals were not obviously increased in the fibrotic group at this time point (Figure S1F,G). In contrast, liver stiffness remained elevated, with increased α‐smooth muscle actin (α‐SMA) and collagen signals consistent with a fibrotic, stiff extracellular matrix (ECM) background (Figure S1H,I). No spontaneous tumor lesions were observed in fibrotic livers without tumor inoculation during the experimental window (Figure S1J).

When pancreatic cancer cells (Pan02) were injected into mice with low or high‐stiffness livers (as outlined in Figure 1B), the high‐stiffness group developed a greater metastatic burden, as shown by gross liver appearance and histological evaluation (Figure 1C,D). Quantification showed increased liver/body weight ratio and larger tumor area per section in the high‐stiffness condition (Figure 1E). Masson's trichrome staining further indicated greater collagen deposition within metastatic lesions in stiff livers (Figure 1D,E). Consistently, Ki67 and CK19 staining supported increased tumor growth, while α‐SMA staining highlighted enhanced stromal activation around lesions in the high‐stiffness group (Figure 1F). Together, these data link liver stiffening with pancreatic cancer liver metastasis in patients and show that a fibrotic, stiff liver microenvironment promotes hepatic colonization and outgrowth in mice under conditions controlled for overt CCl_4_‐driven injury or inflammation.

### 2.2 Matrix Stiffness Activates HSCs Through Piezo1‐Mediated Ca^2^^+^ Signaling

**FIGURE 2:**
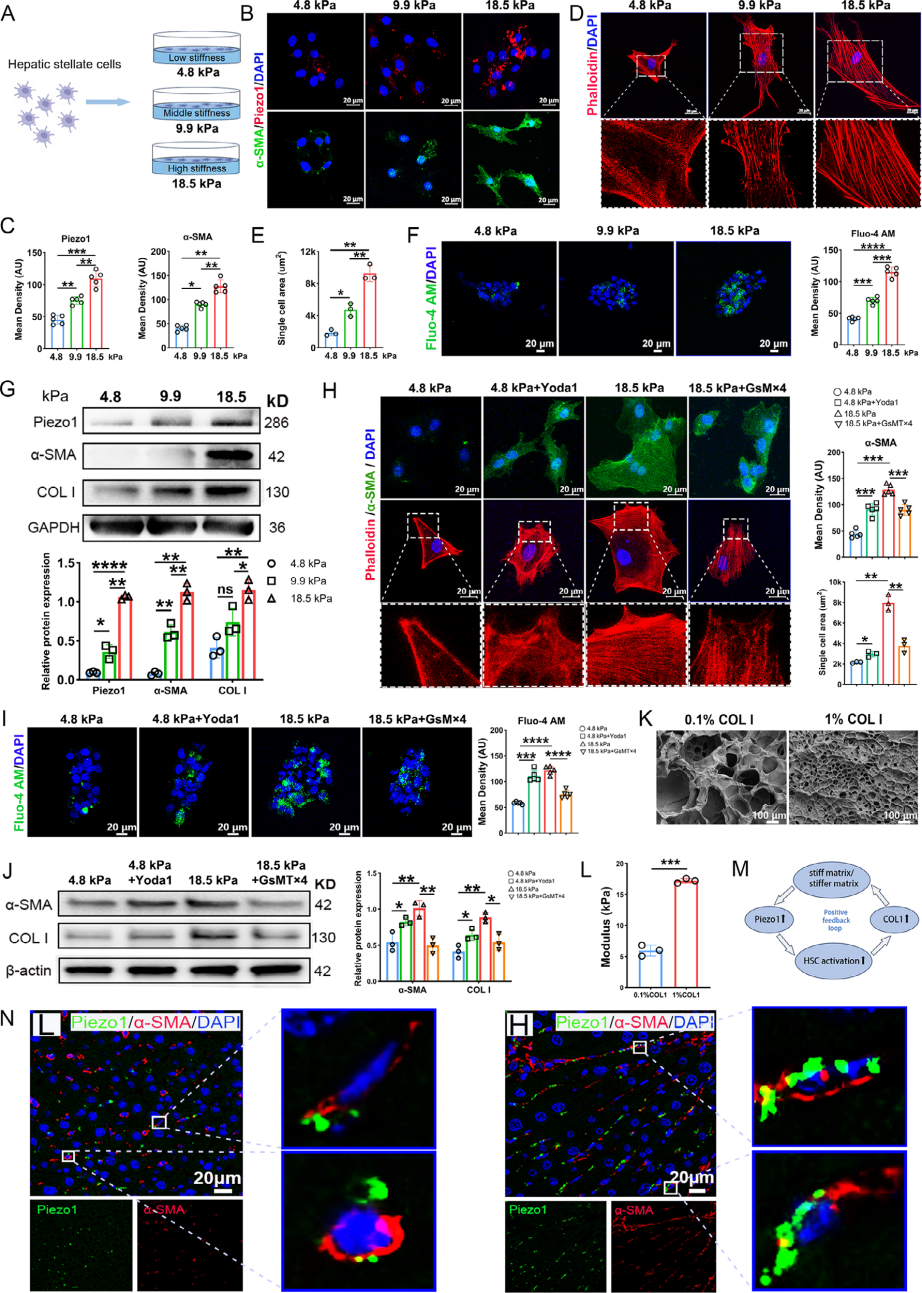
Matrix stiffness activates hepatic stellate cells via Piezo1‐dependent Ca^2^^+^ influx and reinforces a collagen‐deposition positive‐feedback loop. (A) Schematic of culturing hepatic stellate cells (HSCs) on gelatin methacryloyl (GelMA) hydrogels with graded stiffness (low/medium/high). (B,C) Representative immunofluorescence images and quantification showing increased α‐SMA and Piezo1 expression in HSCs with rising matrix stiffness. (D,E) Phalloidin staining and quantification of single‐cell spreading area of HSCs cultured on hydrogels of different stiffness (*n* = 3). (F) Representative Fluo‐4 AM imaging and quantification of intracellular Ca^2^^+^ signals in HSCs cultured on hydrogels of different stiffness. (G) Western blotting and densitometric quantification of Piezo1, α‐SMA, and collagen I in HSCs cultured on hydrogels of graded stiffness. (H–J) Effects of the Piezo1 agonist Yoda1 and the mechanosensitive cation channel inhibitor GsMTx4, added during the final 24 h of stiffness conditioning, on α‐SMA/actin cytoskeleton organization (H), Ca^2^^+^ influx (I), and collagen I expression (J) in stiffness‐conditioned HSCs (*n* = 5). (K,L) Scanning electron microscopy and stiffness measurement of low‐stiffness GelMA supplemented with increasing concentrations of collagen I (*n* = 3). (M) Working model illustrating a stiffness–Piezo1–Ca^2^^+^ axis that promotes HSC activation and collagen I deposition, thereby further increasing matrix stiffness. (N) Representative immunofluorescence images showing elevated Piezo1 expression in α‐SMA^+^ cells in high‐stiffness livers in vivo. Scale bars are indicated in the images. All data are presented as mean ± SD. Statistical analysis was performed using two‐tailed Student's t‐test (two groups) or one‐way ANOVA. *p < 0.05, **p < 0.01, ***p < 0.001, ****p < 0.0001.

To model the stiffness changes observed in fibrotic livers, we established a stiffness‐tunable gelatin methacryloyl (GelMA) hydrogel platform and cultured hepatic stellate cells (HSCs) on low (4.82 ± 0.20 kPa), medium (9.86 ± 0.79 kPa), and high stiffness (18.49 ± 0.03 kPa) substrates (Figure 2A and Figure S2A). Unless otherwise indicated, HSCs were cultured on these hydrogels for 5 days before endpoint analyses. The selected hydrogels showed good cytocompatibility by live/dead staining and stable gelation/mechanical behavior by rheological testing (Figure S2B,C), supporting their use for probing stiffness‐driven HSC responses.

On this platform, increasing matrix stiffness progressively promoted HSC activation. Immunofluorescence staining showed stepwise increases in α‐smooth muscle actin (α‐SMA) and Piezo1 signals from low to high stiffness, which was confirmed by semi‐quantification (Figure 2B,C). Stiffer matrices also enhanced actin stress fiber formation and increased single‐cell spreading area (Figure 2D,E). Consistent with Piezo1 activation, Fluo‐4 AM imaging revealed stronger intracellular Ca^2^^+^ signals in HSCs on stiffer substrates (Figure 2F). Western blotting further supported these observations, showing increased Piezo1, α‐SMA, and collagen I levels with increasing stiffness (Figure 2G).

We next tested whether mechanosensitive cation channel activity is required for the stiffness‐induced phenotype. Cells were treated with Yoda1 or GsMTx4 for 24 h before imaging or protein collection. Pharmacological activation with Yoda1 (Piezo1 agonist) increased α‐SMA signal and cell spreading on the low‐stiffness substrate, whereas inhibition with GsMTx4 reduced α‐SMA and reversed the enlarged spreading morphology on the high‐stiffness substrate (Figure 2H). In parallel, Yoda1 increased Ca^2^^+^ signals, while GsMTx4 decreased the stiffness‐associated Ca^2^^+^ influx (Figure 2I). Western blotting confirmed that Yoda1 enhanced, whereas GsMTx4 attenuated, α‐SMA and collagen I expression under the corresponding conditions (Figure 2J). These results support a model in which stiffness‐driven HSC activation depends on mechanosensitive cation channel‐mediated Ca^2^^+^ entry.

Because activated HSCs deposit collagen, we next examined whether collagen accumulation further stiffen the microenvironment and reinforce HSC activation. Adding collagen I to low‐stiffness GelMA increased matrix density by scanning electron microscopy and markedly increased the measured modulus (Figure 2K,L), consistent with collagen‐dependent stiffening. Together with the Piezo1/Ca^2^^+^‐dependent activation data, these findings support a positive‐feedback loop in which stiffness activates HSCs, HSCs deposit collagen, and collagen further increases matrix stiffness (Figure 2M). In vivo, Piezo1 signal was more prominent in α‐SMA‐positive cells in stiff livers than in soft livers, aligning with the in vitro findings (Figure 2N).

Finally, genetic suppression of Piezo1 supported the pharmacological results. Piezo1 mRNA increased with substrate stiffness (Figure S3A), and Yoda1/GsMTx4 modulated fibrosis‐related transcripts (COL1A1/COL1A2/LOXL2) in a stiffness‐dependent manner (Figure S3B). Piezo1 knockdown reduced α‐SMA expression and cell spreading under high‐stiffness culture, weakened stiffness‐induced Ca^2^^+^ signals, and decreased α‐SMA and collagen I protein levels (Figure S3C–J). Collectively, these data identify Piezo1‐mediated Ca^2^^+^ influx as a key mechanotransduction route by which matrix stiffness drives HSC activation and collagen‐dependent matrix stiffening.

### 2.3 Stiffness‐educated HSC Secretome Induces Epithelial Features and Lipid Reprogramming in PDAC Cells

**FIGURE 3:**
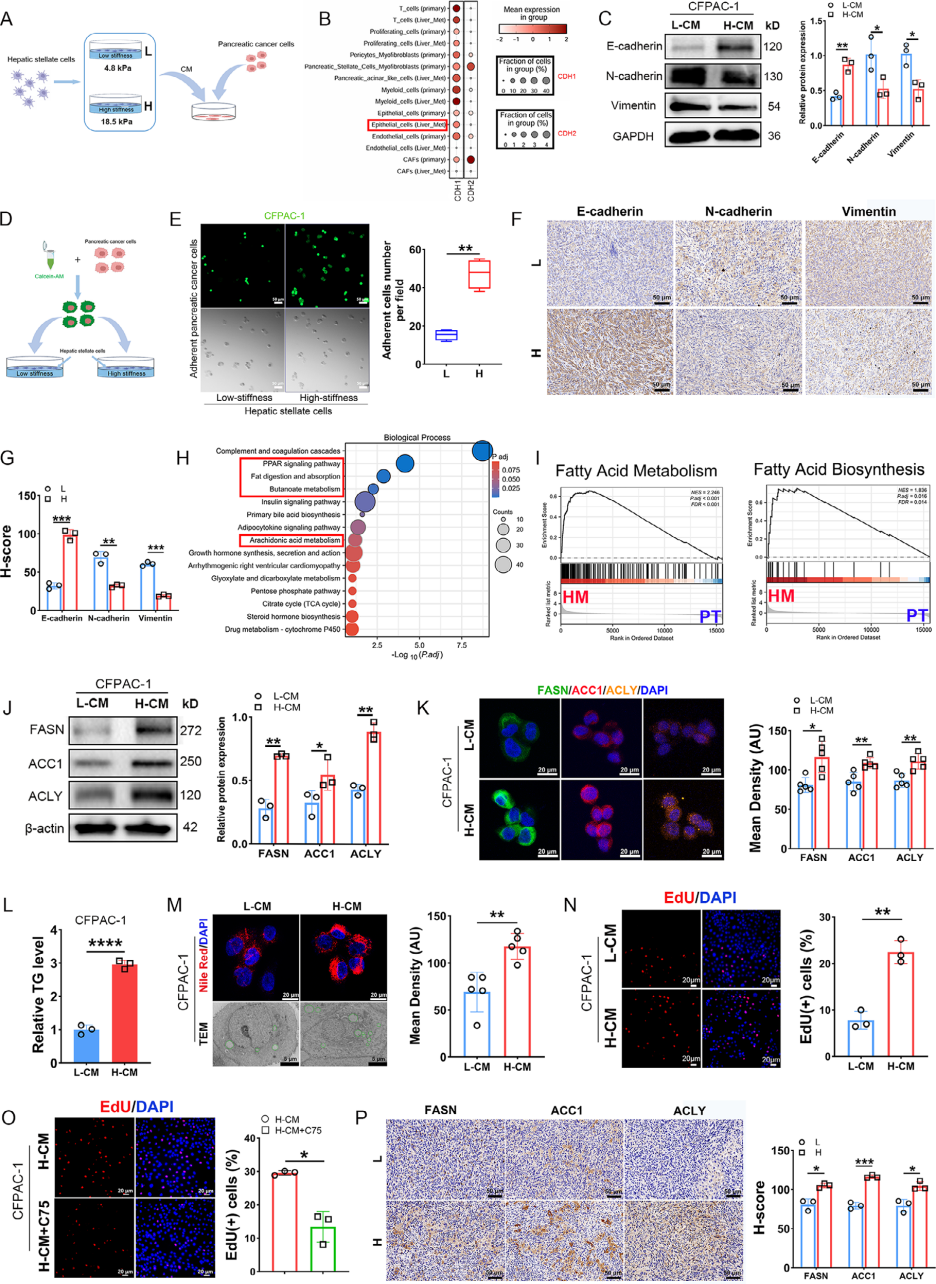
Conditioned medium from stiff‐matrix‐educated HSCs induces a MET‐like program and lipid anabolism in pancreatic cancer cells. (A) Schematic of preparing conditioned medium from HSCs cultured on low‐stiffness (L) or high‐stiffness (H) matrices and treating pancreatic cancer cells with low‐stiffness conditioned medium (L‐CM) or high‐stiffness conditioned medium (H‐CM). (B) Analysis of dataset GSE154778 comparing primary PDAC and liver metastasis samples, showing higher CDH1 (E‐cadherin) expression and a relative CDH1/CDH2‐high pattern in the liver metastasis group. (C) Western blotting and quantification of epithelial/mesenchymal markers in CFPAC‐1 cells following H‐CM treatment (*n* = 3). (D,E) Representative adhesion assay images and quantification showing enhanced adhesion of pancreatic cancer cells after H‐CM treatment (*n* = 4). (F,G) Representative IHC staining and H‐score quantification of E‐cadherin, N‐cadherin, and vimentin in liver metastatic lesions from L and H groups in vivo (*n* = 3). (H) KEGG pathway enrichment analysis of liver metastasis–associated transcriptomic data (dataset GSE34153). (I) Gene set enrichment analysis (GSEA) of dataset GSE34153 comparing PDAC liver metastases with primary pancreatic tumors, showing enrichment of lipid metabolic programs in the liver metastasis group. (J, K) Western blotting and immunofluorescence analyses (with quantification) of key lipogenic enzymes fatty acid synthase (FASN), acetyl‐CoA carboxylase 1 (ACC1), and ATP‐citrate lyase (ACLY) in pancreatic cancer cells treated with H‐CM (*n* = 3). (L) Intracellular triglyceride (TG) quantification after L‐CM or H‐CM treatment (*n* = 3). (M) Nile Red staining and quantitative analysis of neutral lipid droplets in cancer cells after H‐CM treatment (*n* = 5). (N) EdU incorporation assay and quantification showing increased proliferation after H‐CM treatment (*n* = 3). (O) Effects of the FASN inhibitor C75 on H‐CM–enhanced proliferation (EdU) with quantification (*n* = 3). (P) Representative IHC images and H‐score quantification of FASN/ACC1/ACLY in liver metastatic lesions from L and H groups. Scale bars are indicated in the images (*n* = 3). All data are presented as mean ± SD. Statistical analysis was performed using two‐tailed Student's t‐test (two groups) or one‐way ANOVA. *p < 0.05, **p < 0.01, ***p < 0.001, ****p < 0.0001.

To determine whether stiffness‐activated hepatic stellate cells (HSCs) instruct pancreatic cancer cells through secreted factors, we collected conditioned medium from HSCs cultured on low‐ or high‐stiffness hydrogels (L‐CM and H‐CM) and applied it to pancreatic cancer cells (Figure 3A). Specifically, HSCs were stiffness‐conditioned for 5 days and after which the supernatant was collected as L‐CM or H‐CM. Analysis of the public cohort GSE154778 showed that liver metastases exhibited higher CDH1 expression than primary tumors, with a CDH1‐high pattern in metastasis (Figure 3B). Consistent with this observation, H‐CM increased E‐cadherin and reduced N‐cadherin/vimentin in PDAC cells, as shown by immunoblotting (Figure 3C and Figure S4E) and further supported by qPCR and immunofluorescence in both cell lines (Figure S4A–D). Functionally, H‐CM significantly enhanced cancer cell adhesion to the matrix (Figure 3D,E and Figure S4J). In parallel, H‐CM reduced motility‐associated behaviors, as indicated by wound‐healing and transwell assays (Figure S4F–I), supporting a shift toward an epithelial‐like, adhesion‐favored state after conditioning by stiffness‐activated HSCs.

We next asked whether the stiffness‐educated HSC secretome also drives metabolic adaptation relevant to metastatic outgrowth. KEGG enrichment analysis of transcriptomic differences between PDAC liver metastases and primary pancreatic tumors highlighted lipid metabolism‐related programs (Figure 3H). GSEA of an independent dataset (GSE34153) further showed enrichment of lipid metabolism‐related gene sets in the liver metastasis group (Figure 3I). Single‐cell analyses further supported the enrichment of lipid‐related signatures and elevated FASN expression in metastatic contexts (Figure S5A–C). In vitro, H‐CM increased levels of key de novo lipogenic enzymes, including fatty acid synthase (FASN), acetyl‐CoA carboxylase 1 (ACC1), and ATP‐citrate lyase (ACLY), in CFPAC‐1 cells (Figure 3J,K). A similar pattern was observed in MIA‐PaCa2 cells by immunoblotting and immunofluorescence (Figure S6A–C), and was also supported at the transcript level (Figures S5D and S6F). Consistent with enhanced lipid anabolism, H‐CM increased intracellular triglyceride levels (Figure 3L and Figure S6D) and promoted neutral lipid droplet accumulation by Nile Red staining (Figure 3M), which was further corroborated in MIA‐PaCa2 cells and by ultrastructural evidence of lipid droplet enrichment in TEM images (Figure S6E). This lipogenic shift was accompanied by increased proliferative activity. EdU incorporation increased in H‐CM treated cells (Figure 3N and Figure S6G), and both colony formation and CCK‐8 assays supported a growth advantage (Figures S5E–H and S6H,I). Importantly, pharmacological inhibition of FASN (C75) attenuated the H‐CM‐associated proliferation gain (Figure 3O) and similarly suppressed growth phenotypes in independent assays (Figure S6J–L). In vivo, metastatic lesions arising in stiff livers showed higher expression of FASN/ACC1/ACLY compared with lesions in soft livers (Figure 3P), aligning with the in vitro lipogenic program induced by H‐CM.

### 2.4 Stiffness Elevates HSC‐Derived GMFG via Piezo1‐Ca^2^^+^‐IRE1α/XBP1 Signaling

**FIGURE 4:**
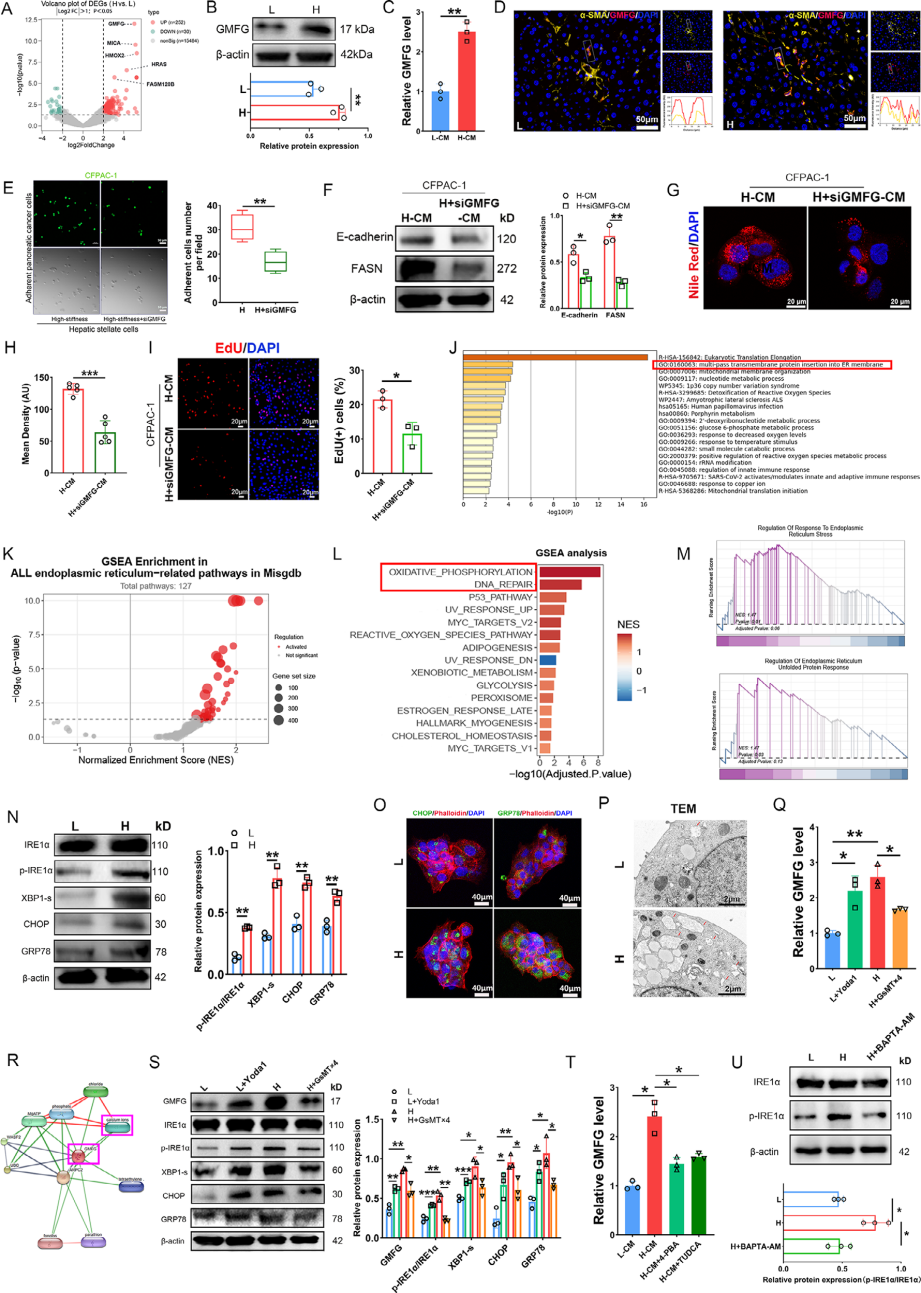
Piezo1‐driven endoplasmic reticulum stress in stiff‐matrix–activated HSCs promotes GMFG secretion and reprograms pancreatic cancer cells. (A) Volcano plot of RNA‐seq differential expression in HSCs cultured on low‐ versus high‐stiffness matrices. (B) Western blotting and quantification showing increased glia maturation factor gamma (GMFG) expression in high‐stiffness–cultured HSCs (*n* = 3). (C) ELISA quantification of secreted GMFG in conditioned media collected from low‐ and high‐stiffness HSC cultures (*n* = 3). (D) Representative immunofluorescence images showing co‐localization of α‐SMA and GMFG in vivo with line‐scan analysis. (E–I) Functional validation using GMFG‐silenced high‐stiffness HSCs: adhesion assay (*n* = 4) (E), Western blotting of E‐cadherin and FASN (*n* = 3) (F), Nile Red staining of lipid droplets (n = 5) (G,H), and EdU proliferation assay (*n* = 3) (I) in pancreatic cancer cells treated with H‐CM or GMFG‐deficient H‐CM. (J) Metascape enrichment analysis of RNA‐seq differential genes. (K–M) GSEA of endoplasmic reticulum (ER)‐related gene sets in HSCs cultured on high‐ versus low‐stiffness matrices, with representative enrichment plots indicating activation of ER stress/unfolded protein response pathways in the high‐stiffness group. GSEA was performed using MSigDB Hallmark gene sets. (N–P) ER stress validation in stiffness‐conditioned HSCs by Western blotting of ER stress markers (*n* = 3) (N), immunofluorescence (O), and transmission electron microscopy (TEM) (P). (Q) ELISA showing that Yoda1 and GsMTx4 modulate GMFG secretion, consistent with Piezo1‐mediated Ca^2^^+^ entry (*n* = 3). (R) Protein–chemical/ion association network of GMFG constructed from STITCH. (S) Western blotting showing coordinated regulation of GMFG and ER stress proteins by Yoda1 and GsMTx4 (*n* = 3). (T) Effects of ER stress inhibitors 4‐phenylbutyric acid (4‐PBA) and tauroursodeoxycholic acid (TUDCA) on GMFG secretion from high‐stiffness HSCs (*n* = 3). (U) Effects of the Ca^2^^+^ chelator BAPTA‐AM on stiffness‐associated p‐IRE1α elevation in HSCs. Scale bars are indicated in the images (*n* = 3). All data are presented as mean ± SD. Statistical analysis was performed using two‐tailed Student's t‐test (two groups) or one‐way ANOVA. *p < 0.05, **p < 0.01, ***p < 0.001.

To identify stiffness‐regulated paracrine mediators from HSCs, we performed RNA‐seq comparing HSCs cultured on high‐ versus low‐stiffness substrates (Figure 4A), with clear sample separation supported by principal component analysis (Figure S7A). Among the most strongly induced genes, glia maturation factor gamma (GMFG) emerged as a prominent stiffness‐upregulated candidate (Figure 4A). We validated the increase in GMFG protein abundance by immunoblotting (Figure 4B) and confirmed higher GMFG secretion into conditioned medium by ELISA (Figure 4C). In vivo, GMFG signal co‐localized with α‐SMA–positive stromal cells, and line‐scan analysis supported their spatial overlap in stiff livers (Figure 4D). Importantly, the key findings of our study—including stiffness‐induced HSC activation, increased GMFG synthesis and secretion, and transmission of pro‐adhesive/pro‐lipogenic effects to pancreatic cancer cells—were reproduced in primary murine HSCs.

We then tested whether HSC‐derived GMFG is required for H‐CM‐driven phenotypes in pancreatic cancer cells. GMFG knockdown efficiency in HSCs was confirmed (Figure S7B). Depleting GMFG from H‐CM reduced cancer cell adhesion compared with control H‐CM (Figure 4E), which was reproduced in an additional adhesion dataset (Figure S7C). In parallel, GMFG‐deficient H‐CM blunted the H‐CM‐induced increase of E‐cadherin and FASN in CFPAC‐1 cells (Figure 4F). Consistently, GMFG‐deficient H‐CM also reduced epithelial and lipogenic readouts in supporting validation experiments (Figure S7D,E). Consistent with this, GMFG depletion decreased lipid droplet accumulation (Figure 4G,H and Figure S7F) and reduced proliferative outputs (Figure 4I and Figure S7G). These results indicate that GMFG is a major stiffness‐encoded, HSC‐derived effector that drives adhesion and lipogenic growth programs in pancreatic cancer cells.

Mechanistically, enrichment analyses of stiffness‐regulated genes pointed to pathways related to endoplasmic reticulum stress (ERS) and the unfolded protein response (UPR) (Figure 4J), and curated ER‐related gene sets were used for GSEA (Figure 4K). GSEA consistently indicated activation of ER‐associated programs under high stiffness (Figure 4L,M). ERS activation was verified by immunoblotting of ERS markers (including p‐IRE1α and downstream signals) (Figure 4N), with additional immunoblot validation shown in Figure S7J. Importantly, cell‐level evidence of ERS activation was provided by Figure S7I. Immunofluorescence staining of CHOP and GRP78 showed increased ERS marker signals under high stiffness and their reduction after ERS modulation, supporting that stiffness induces a bona fide ER stress response in HSCs rather than a purely transcriptional signature. ER ultrastructural alterations consistent with ER stress were further observed by TEM (Figure 4P), and ERS‐associated transcripts were also increased at the mRNA level (Figure S7K). To link mechanosensing to ER stress, organelle‐probe imaging showed altered Ca^2^^+^ distribution between ER and mitochondria under stiffness/Piezo1 modulation (Figure S7L), supporting a Ca^2^^+^‐coupled mechanism.

We next linked Piezo1‐mediated Ca^2^^+^ entry to GMFG output. Pharmacological Piezo1 activation with Yoda1 increased GMFG secretion, whereas mechanosensitive cation channel inhibition (GsMTx4) reduced it (Figure 4Q). These interventions produced coordinated changes in ERS markers and GMFG expression (Figure 4S), consistent with a Piezo1‐Ca^2^^+^‐ERS linkage; the GMFG‐centered interaction network is shown in Figure 4R. Blocking ER stress using 4‐phenylbutyric acid (4‐PBA) or tauroursodeoxycholic acid (TUDCA) reduced GMFG secretion (Figure 4T), and chelating intracellular Ca^2^^+^ with BAPTA‐AM attenuated stiffness‐associated IRE1α phosphorylation (Figure 4U), supporting a Piezo1‐Ca^2^^+^‐IRE1α/XBP1 axis controlling GMFG production. Recombinant GMFG add‐back experiments showed sufficiency: supplementing L‐CM with recombinant human GMFG (rGMFG) shifted epithelial marker patterns (Figure S9A), enhanced adhesion (Figure S9B,C), increased lipogenic enzymes (Figure S9D), elevated lipid droplet accumulation and triglyceride levels (Figure S9E–H), and promoted proliferative outputs (Figure S9I), collectively recapitulating key H‐CM‐associated phenotypes.

To clarify how HSC‐derived extracellular GMFG is delivered to pancreatic cancer cells, we fractionated H‐CM into EV and EV‐free fractions. Transmission electron microscopy, particle size analysis, and EV marker detection confirmed successful enrichment of EVs (Figure S10A–C). Notably, GMFG was predominantly detected in the EV fraction. Functionally, the EV fraction retained strong pro‐adhesive and pro‐lipogenic activity, whereas the EV‐free fraction exerted substantially weaker effects. Moreover, EVs isolated from GMFG‐silenced HSCs displayed markedly attenuated ability to promote pancreatic cancer cell adhesion, E‐cadherin/FASN regulation, and lipid droplet accumulation (Figure S10D–F). These data indicate that HSC‐derived GMFG is transported, at least in part, in an EV‐associated extracellular form and functionally delivered to recipient tumor cells.

### 2.5 GMFG Binds TNS4 to Activate FAK‐AKT Signaling and Drive Adhesion and Lipid Reprogramming

**FIGURE 5:**
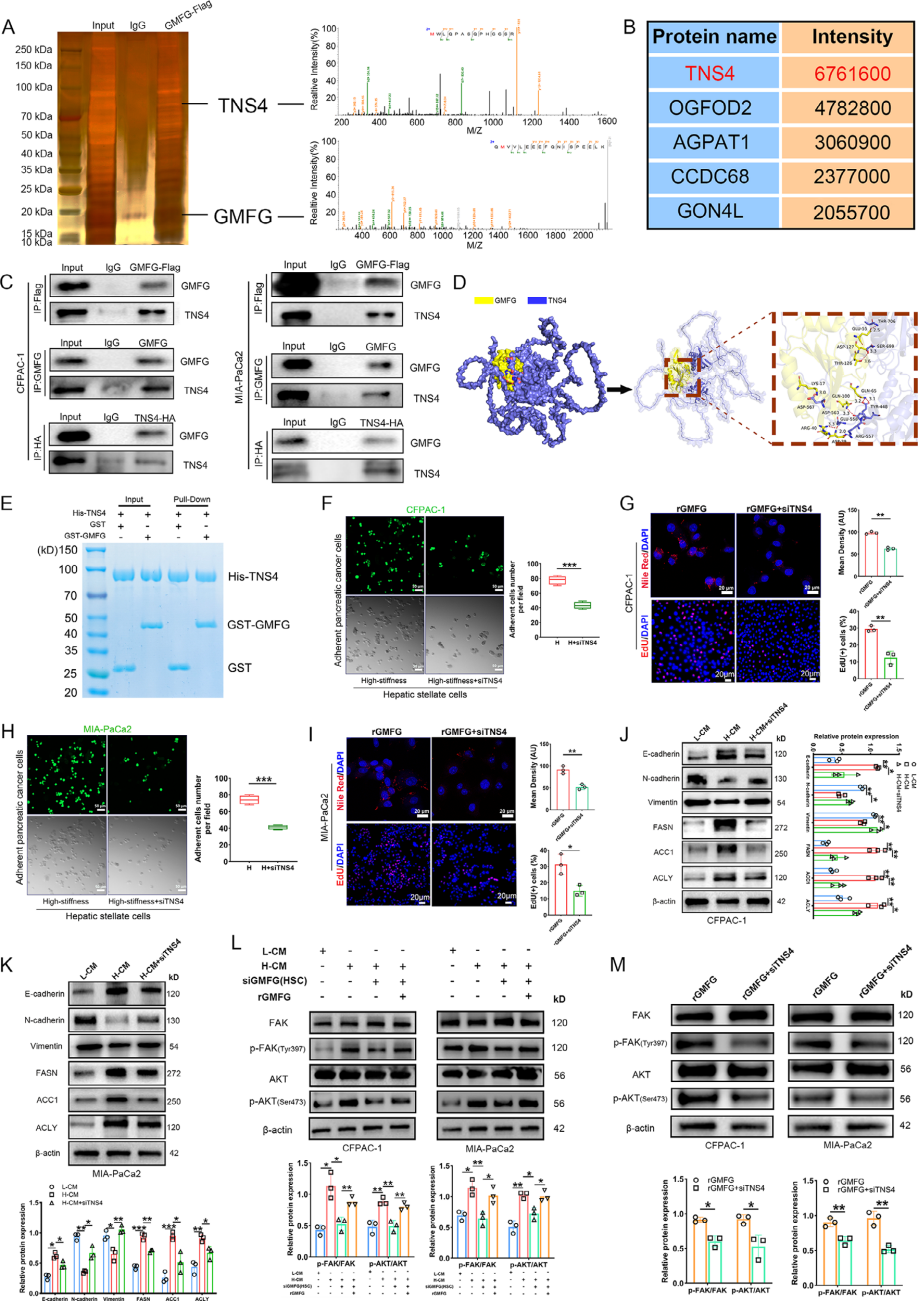
Paracrine GMFG engages TNS4 to activate FAK/AKT signaling and drive adhesion and lipogenic growth programs in pancreatic cancer cells. (A) Anti‐Flag immunoprecipitation of GMFG‐Flag followed by silver staining and mass spectrometry to identify GMFG‐associated proteins. (B) Ranking of the top enriched co‐precipitated proteins in the GMFG‐Flag group. (C) Reciprocal co‐immunoprecipitation validating the interaction between GMFG and tensin 4 (TNS4). (D) Protein–protein docking model illustrating a stable GMFG–TNS4 binding interface. (E) Coomassie blue staining verifying the expected integrity and molecular weight of purified GST‐tagged proteins used for pull‐down. (F,H) Adhesion assays and quantification showing reduced adhesion of CFPAC‐1 (F) and MIA‐PaCa2 (H) cells after small interfering RNA (siRNA)‐mediated knockdown of TNS4 (*n* = 4). (G,I) Nile Red staining (*n* = 5) and EdU assays (with quantification) (*n* = 3) showing that recombinant human GMFG (rGMFG)–induced lipid droplet accumulation and proliferation are attenuated by TNS4 knockdown. (J,K) Western blotting and quantification showing that H‐CM–induced upregulation of E‐cadherin and lipogenic enzymes (FASN/ACC1/ACLY) is suppressed by TNS4 knockdown in CFPAC‐1 (J) and MIA‐PaCa2 (K) cells (*n* = 3). (L,M) Western blotting showing increased phosphorylation of focal adhesion kinase (FAK, Y397) and AKT (S473) upon 48 h H‐CM or rGMFG stimulation, which is diminished by GMFG depletion in HSCs and/or TNS4 knockdown in cancer cells, with rGMFG add‐back as indicated (*n* = 3). Scale bars are indicated in the images. All data are presented as mean ± SD. Statistical analysis was performed using two‐tailed Student's t‐test (two groups) or one‐way ANOVA. *p < 0.05, **p < 0.01, ***p < 0.001.

We next investigated how extracellular GMFG transmits signals in pancreatic cancer cells. To identify GMFG‐associated proteins, we performed anti‐Flag immunoprecipitation of GMFG‐Flag followed by silver staining and mass spectrometry, which consistently identified tensin‐4 (TNS4) as a prominent GMFG‐interacting candidate (Figure 5A,B). The GMFG–TNS4 interaction was further validated by reciprocal co‐immunoprecipitation (Figure 5C), and docking analysis supported a compatible binding interface (Figure 5D). Consistent with clinical relevance, single‐cell analysis (GSE154778) showed enrichment of GMFG/TNS4 signals within epithelial tumor cell populations (Figure S11A). To further verify the physical and spatial association between GMFG and TNS4, we performed GST pull‐down assays and fluorescence uptake/co‐localization analysis. GST pull‐down assays supported a direct interaction between the two proteins (Figure 5E and Figure S11B). In parallel, red‐labeled recombinant GMFG showed progressive punctate intracellular accumulation in pancreatic cancer cells and displayed partial spatial overlap with TNS4, consistent with intracellular localization (Figure S11D). Together with proximity ligation assay data (Figure S11C), these results reinforce that extracellular GMFG can reach recipient tumor cells and physically and functionally engage the TNS4 axis. Functionally, TNS4 knockdown reduced matrix adhesion of pancreatic cancer cells and attenuated the adhesion‐promoting effect induced by GMFG/H‐CM, as demonstrated in both CFPAC‐1 and MIA‐PaCa2 cells (Figure 5F,H). In addition, TNS4 depletion weakened GMFG‐associated lipid droplet accumulation and proliferative outputs (Figure 5G,I). Epithelial marker (Figure S12B–E) and lipogenic enzymes (Figure S12F) were reduced by immunofluorescence and triglyceride levels decreased upon TNS4 depletion (Figure S12G). Growth suppression was further supported by CCK‐8 and colony formation assays (Figure S12H,I). Importantly, spatial transcriptomics analysis of pancreatic cancer liver metastases showed spatial enrichment of GMFG and XBP1 signals and their alignment with fatty acid metabolism/lipogenesis‐associated programs within metastatic regions (Figure S12J), supporting an in situ link between the upstream ER‐stress/GMFG module and tumor lipogenic states.

To gain insight into pathways downstream of TNS4, we performed a pan‐cancer correlation analysis and selected a robust gene set that remained positively correlated with TNS4 across ≥20 cancer types (cutoff determination shown in Figure S13A). Gene ontology (GO) enrichment of this robust gene set highlighted terms related to cell‐substrate junctions, focal adhesion, and integrin binding (Figure S13B). KEGG enrichment similarly indicated significant enrichment of Focal adhesion and Integrin signaling (Figure S13C). These results suggested that TNS4 is closely linked to the focal‐adhesion/integrin signaling module, guiding our subsequent mechanistic validation of the core FAK‐AKT axis.

Given the focal adhesion linkage indicated by pan‐cancer enrichment, we next examined the FAK‐AKT pathway. H‐CM or recombinant GMFG increased FAK and AKT phosphorylation, whereas GMFG depletion in HSCs and TNS4 knockdown in tumor cells reduced this phosphorylation response (Figure 5L,M). Together, these results support a model in which extracellular GMFG engages intracellular TNS4 to activate FAK‐AKT signaling, thereby coordinating enhanced cell‐ECM adhesion and lipid reprogramming in pancreatic cancer cells.

### 2.6 Inhibiting Mechanosensitive Cation Channels and Silencing Tumor‐Cell Tns4 Suppress Hepatic Metastatic Progression and Early Seeding

**FIGURE 6:**
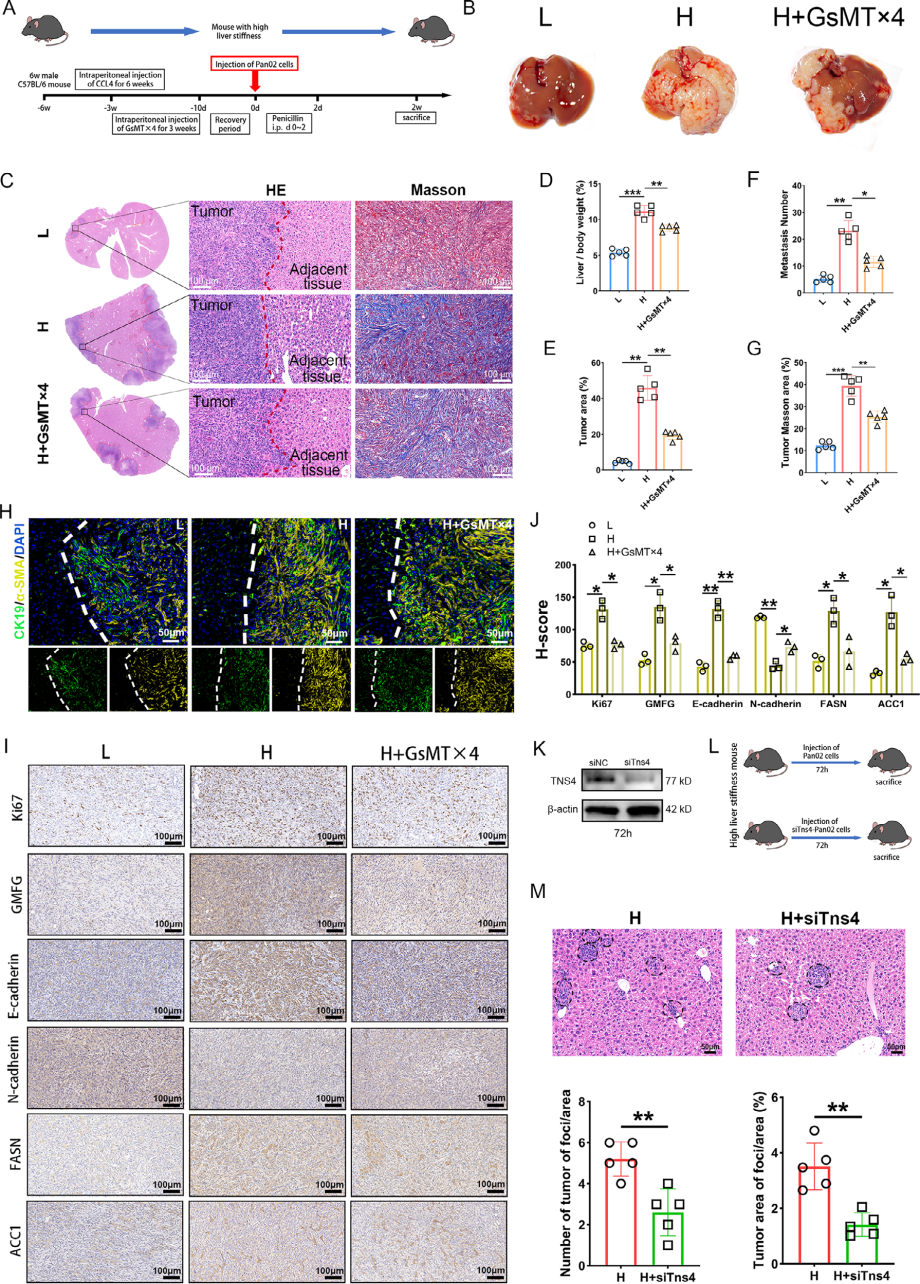
Pharmacological blockade of mechanosensitive cation channels mitigates liver stiffness–enhanced metastatic progression and early colonization in vivo. (A) Experimental scheme for generating high‐stiffness livers and administering the mechanosensitive cation channel inhibitor GsMTx4 during the fibrotic phase before/around tumor inoculation. (B) Representative images of livers from indicated groups. (C) Representative H&E and Masson's trichrome staining of metastatic lesions and adjacent liver tissues. (D–G) Quantification of liver‐to‐body weight ratio, metastatic burden (number of metastatic foci), tumor area ratio, and Masson‐positive area within tumor regions (*n* = 5). (H) Representative CK19 and α‐SMA immunofluorescence staining showing tumor regions and stromal activation/fibrotic response (dashed lines indicate tumor boundaries). (I,J) Representative IHC images and H‐score quantification for Ki67, GMFG, E‐cadherin, N‐cadherin, FASN, and ACC1 in liver metastatic lesions from indicated groups (*n* = 3). (K) Western blotting verification of siRNA‐mediated knockdown efficiency of TNS4 in Pan02 cells (72 h). (L) Schematic of injecting siRNA‐treated Pan02 cells into the high‐stiffness liver background and harvesting tissues at 72 h for early colonization assessment. (M) Representative H&E images of early metastatic foci with quantification of foci number and area (*n* = 5). Scale bars are indicated in the images. All data are presented as mean ± SD. Statistical analysis was performed using two‐tailed Student's t‐test (two groups) or one‐way ANOVA. *p < 0.05, **p < 0.01, ***p < 0.001.

We next tested whether blocking mechanosensitive cation channels could suppress liver stiffness‐enhanced metastatic progression in vivo. In the stiffness‐graded liver model, mice in the high‐stiffness (H) group were treated with GsMTx4 as outlined (Figure 6A). Compared with untreated H mice, the H+GsMTx4 group showed a clear reduction in liver metastatic burden by gross inspection and histological evaluation (Figure 6B,C). Quantification further supported a decreased metastatic load, including a reduced liver/body weight ratio, metastatic foci number, and tumor area in the H+GsMTx4 group (Figure 6D–F). Masson's trichrome staining also indicated decreased collagen deposition within tumor regions after GsMTx4 treatment (Figure 6C,G). Liver stiffness was also reduced (Figure S15). Consistently, CK19/α‐SMA staining suggested attenuated stromal activation surrounding metastatic lesions in the H+GsMTx4 group (Figure 6H). We then examined whether GsMTx4 treatment dampened the downstream tumor signatures associated with the GMFG pathway. Immunohistochemistry showed reduced Ki67 and decreased GMFG‐associated epithelial and lipogenic readouts in metastatic lesions from the H+GsMTx4 group, including changes in E‐cadherin/N‐cadherin and reduced fatty acid synthesis markers (FASN and ACC1) (Figure 6I,J). These results indicate that mechanosensitive cation channel activity is required for stiffness‐driven metastatic outgrowth and associated molecular programs in vivo.

To directly test the contribution of the GMFG‐TNS4 axis during early colonization, we silenced Tns4 in Pan02 cells before inoculation. Knockdown efficiency was confirmed by immunoblotting and qPCR (Figure 6K and Figure S14). When siNC‐ or siTns4‐treated Pan02 cells were injected into the stiff‐liver background and livers were collected at 72 h (Figure 6L), Tns4 depletion significantly reduced early hepatic micrometastatic seeding, as assessed by H&E staining and quantification of early foci number and area (Figure 6M). Together, these data support that mechanosensitive cation channel inhibition suppresses stiffness‐enhanced metastatic progression and that tumor‐cell TNS4 is required for efficient early establishment in the stiff hepatic niche.

Finally, to further complement the pharmacological GsMTx4 experiments with a more precise and durable downstream genetic intervention, we established stable Tns4 knockdown Pan02 cells and used them in the stiff‐liver metastasis model (Figure S16A,B). Compared with shNC controls, Tns4 knockdown significantly reduced liver/body weight ratio, metastatic nodule number, tumor area, and intratumoral collagen deposition at the defined 2‐week endpoint (Figure S16C,D). These data further demonstrate that the downstream GMFG–TNS4 arm is functionally required under stiff‐liver conditions.

## 3 Discussion

Liver stiffening should be viewed not only as a physical attribute of the metastatic “soil” but also as an instructive and dynamically amplifiable niche signal that can be propagated across cell types. Recent syntheses of pre‐metastatic niche (PMN) biology emphasize that “soil” signals are not purely biochemical but emerge from an integrated, multicellular program in which stromal remodeling, immune education, angiogenesis, and tissue mechanics co‐evolve to bias organotropism [36, 37, 38, 39]. Building on clinical observations of locally stiffened hepatic regions surrounding metastatic lesions, we propose that fibrosis‐associated mechanics contribute to PDAC liver colonization through two coordinated routes: direct tumor cell‐autonomous mechanosensing and an HSC‐mediated paracrine amplification program. Clinically, lesion stiffness measured by elastography has been reported to associate with poorer outcomes in pancreatic cancer patients with liver metastasis, supporting stiffness as a quantifiable risk feature rather than a purely experimental construct [17]. While tumor‐intrinsic mechanotransduction in PDAC has been extensively documented, our work highlights a complementary stromal mechanism in which mechanical stress first reprograms HSCs and thereby converts a biomechanical cue into a soluble, targetable instruction that shapes tumor behavior. This “mechanics‐stromal‐statesoluble instruction” framing is increasingly recognized as a generalizable PMN logic, particularly for fibroblast‐lineage cells that can amplify local cues into organ‐wide permissive niches [36, 40].

A key conceptual feature of this model is the existence of a feed‐forward amplification loop. Increased matrix stiffness activates HSCs, promoting further extracellular matrix deposition and remodeling, which in turn reinforces tissue stiffening and mechanosensitive signaling. Such stiffness fibroblast reciprocity is now viewed as a core mechanism by which localized fibrotic microdomains stabilize and expand, providing a physical explanation for spatially clustered colonization “hotspots” [41]. Mechanistically, we position mechanosensitive Ca^2^^+^ entry and ER stress signaling as a plausible transduction module that couples HSC mechanosensing to a secretory output. Accumulating evidence indicates that plasma‐membrane tension and mechanotransduction can be relayed to intracellular organelles (including the ER) through membrane contact sites, providing a concrete physical route for mechanics‐to‐ER stress coupling [42]. In the liver context, Piezo1 is increasingly implicated as a mechanosensitive regulator in hepatic disease states [43].

Although GMFG has traditionally been regarded as an intracellular protein, our findings indicate that it can be released into the extracellular space in an environment‐dependent manner through exosome‐associated secretion linked to stress‐adaptive responses. Notably, stress‐adaptive, Golgi‐bypass unconventional protein secretion (UcPS) has been highlighted as a common route by which leaderless cytosolic proteins gain extracellular functions under cellular stress [44, 45]. A recent Advanced Science study further provides direct precedent that stromal cells can engage paracrine GMFG signaling, supporting the feasibility of GMFG functioning as an extracellular instruction in specific stress contexts [46]. In this framework, ER stress extends beyond tumor‐intrinsic adaptation and may function within stromal cells as an upstream “signal generator” that translates biomechanical perturbation into paracrine instruction.

Importantly, our data support a functional link between extracellular GMFG and TNS4‐dependent adhesion signaling in tumor cells. Specifically, stiffness‐activated HSCs release GMFG in an EV‐associated extracellular form, which is subsequently taken up by pancreatic cancer cells and functionally engages the TNS4 axis to promote adhesion, FAK/AKT activation, and lipogenic reprogramming. Rather than assuming direct cytosolic transfer, our model emphasizes functional engagement of a focal‐adhesion module downstream of TNS4. This is consistent with the established role of TNS4/CTEN as a focal‐adhesion scaffold that stabilizes integrin complexes and potentiates FAK‐PI3K/Akt signaling outputs [47, 48]. Future studies dissecting receptor involvement, endocytic routing, or membrane‐proximal interaction dynamics may uncover additional regulatory checkpoints within this communication pathway. In addition, although our bioinformatic analyses suggest that additional stromal or immune populations, including T cells, may also be involved in the metastatic niche, the present study was not designed to functionally dissect their contribution. Therefore, our mechanistic conclusions are restricted to the experimentally validated HSC‐derived GMFG–TNS4 axis.

Downstream, the stroma‐to‐tumor communication described here integrates focal‐adhesion signaling with metabolic rewiring‐two essential requirements for efficient colonization. Instead of interpreting epithelial features, adhesion reinforcement, and lipid synthesis as independent observations, our framework views them as coordinated outputs of a niche‐instructed program. This coordination matches mechanobiology literature in which ECM stiffness is recognized as a regulator of cellular metabolism (including anabolic programs), linking adhesion signaling to bioenergetic and biosynthetic state control [49]. Strengthened cell‐ECM anchorage provides mechanical stability for hepatic seeding, while enhanced de novo fatty acid synthesis supports anabolic growth within the metabolically distinct liver microenvironment. FAK/Src‐family signaling has also been implicated in directly modulating metabolic enzymes and metabolites to support metastatic competence, providing an independent rationale for why adhesion hubs and anabolic metabolism frequently co‐segregate [50]. In this context, E‐cadherin should be interpreted as a marker of epithelial colonization competence rather than over‐attributed to enhanced intercellular adhesion.

Unbiased enrichment analyses consistently highlight integrin/focal‐adhesion processes as central nodes associated with TNS4, offering orthogonal validation for prioritizing the FAK‐AKT axis. More broadly, these findings suggest that metastasis‐promoting stromal signals may converge on adhesion signaling hubs that are intrinsically coupled to anabolic metabolism. This convergence provides a mechanistic rationale for why targeting niche mechanics can simultaneously influence both physical colonization and metabolic fitness. Recent research of tensin family biology underscore that TNS4 often behaves as a “signal amplifier” at adhesions with biomarker potential across carcinomas, reinforcing the translational relevance of centering this node [48]. Given the expanding recognition of integrin/adhesion signaling in therapy resistance, positioning the GMFG–TNS4 axis upstream of these hubs may also help rationalize combination strategies that prevent compensatory escape via adhesion‐mediated survival circuits.

From a translational perspective, the stiffness‐to‐colonization cascade identified here reveals multiple intervention layers. Targeting mechanosensitive signaling may dampen niche amplification; disrupting the GMFG‐TNS4 axis may block stromal instruction; and inhibiting lipogenesis may buffer downstream growth advantages. In parallel with genetic approaches, Piezo1‐focused pharmacology and inhibitor development has accelerated in recent years, offering a realistic path to test “mechanosensing blockade” as an anti‐niche strategy [51, 52]. Importantly, because tumor‐intrinsic mechanosensing likely operates in parallel, stromal loop disruption may provide complementary rather than redundant therapeutic benefit by attenuating both matrix remodeling and paracrine reinforcement. Notably, our in vivo validation was performed at a defined 2‐week endpoint and should therefore be interpreted as evidence for effects on early metastatic colonization/outgrowth rather than formal survival benefit. This model also supports stiffness‐informed risk stratification strategies, such as elastography‐based identification of mechanically permissive hepatic niches, and rational combination approaches pairing mechanosensitive inhibition with blockade of adhesion or metabolic nodes. Our findings should be interpreted in the context of emerging evidence that abnormal hepatic mechanics are clinically relevant in metastatic PDAC. Although direct epidemiological evidence specifically demonstrating a higher incidence of PDAC liver metastasis in patients with established cirrhosis than in those without cirrhosis remains limited, available data consistently support an association between altered hepatic mechanical/metabolic background and metastatic progression [17, 18, 19]. In this regard, our observation of a local perimetastatic stiffness gradient is of particular interest, because it suggests that metastatic lesions are accompanied by marked regional mechanical remodeling rather than merely reflecting a uniformly fibrotic liver. At the same time, Figure 1A should be interpreted as a representative clinical example rather than a cohort‐level causal analysis.

Several limitations warrant acknowledgment. First, upstream intervention in vivo relies on GsMTx4, a broad mechanosensitive channel inhibitor, and does not establish Piezo1 specificity or precise cellular attribution. Genetic or cell type‐restricted approaches will be necessary to refine this axis. Second, although functional dependency on TNS4 and FAK‐AKT signaling is well supported, the molecular topology linking extracellular GMFG to intracellular adhesion machinery requires further clarification. Addressing this spatial interface may uncover additional therapeutic opportunities.

Despite these constraints, the overall framework delineates a biologically coherent and experimentally supported stroma‐tumor mechanotransduction axis. By reframing fibrosis‐associated mechanics as an amplifiable signal network rather than a passive physical barrier, this study provides conceptual and therapeutic groundwork for targeting stromal‐to‐tumor communication to limit hepatic colonization.

**FIGURE 7:**
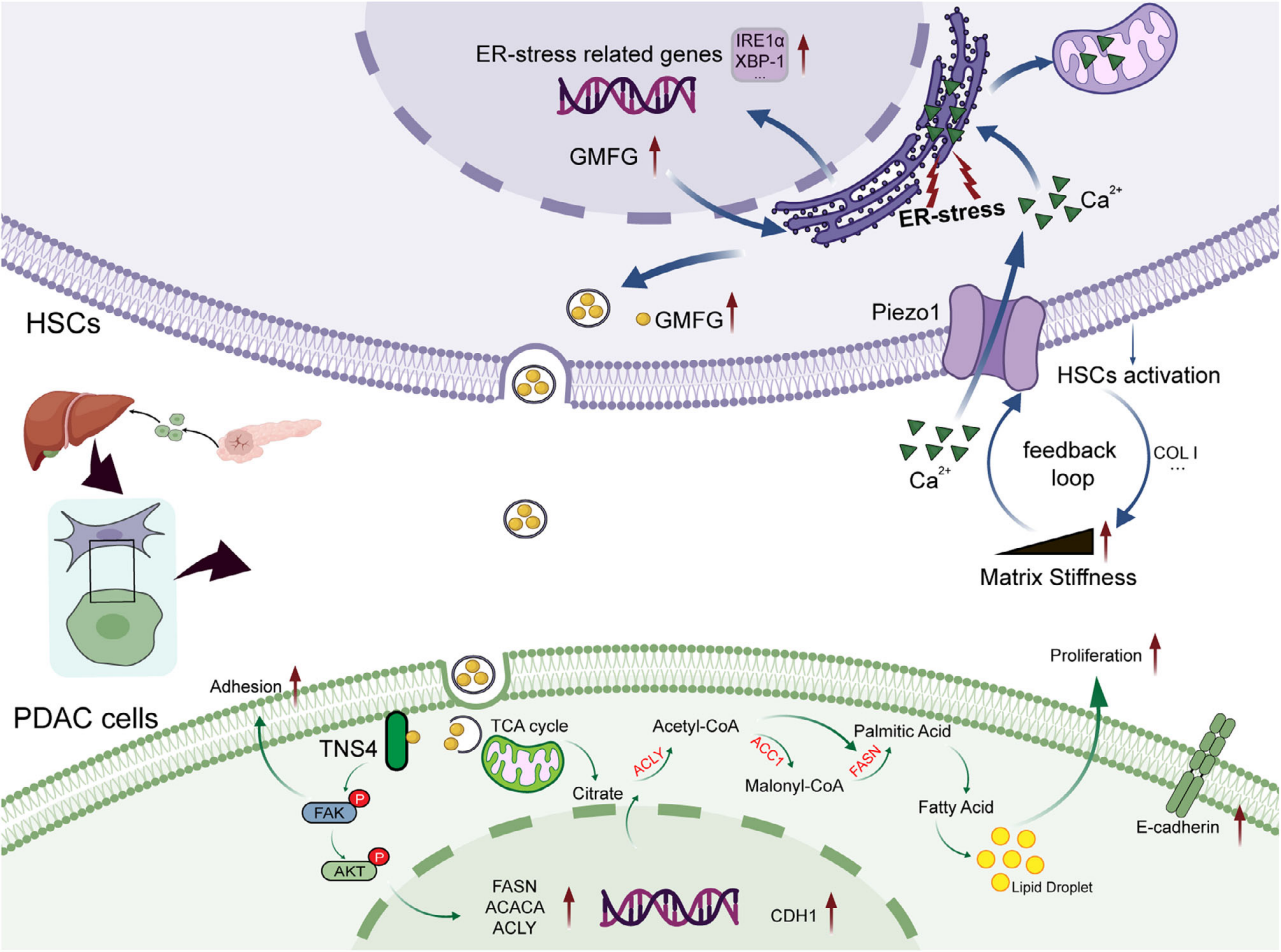
Schematic model of the stiffness–Piezo1–GMFG–TNS4 axis driving pancreatic cancer liver metastasis. A premetastatic niche (PMN) increases liver stiffness, which activates hepatic stellate cells (HSCs) to deposit collagen, further stiffening the extracellular matrix (ECM) and establishing a positive‐feedback loop. Elevated stiffness triggers Piezo1‐mediated Ca^2^^+^ influx, inducing endoplasmic reticulum stress (ERS) and activation of the IRE1α‐XBP1 pathway, thereby upregulating glia maturation factor gamma (GMFG) transcription, synthesis, and secretion. EV‐associated GMFG engages intracellular tensin‐4 (TNS4) in pancreatic cancer cells, promoting FAK and AKT phosphorylation and driving pro‐metastatic phenotypes, including enhanced adhesion and fatty acid synthesis.

In summary, we propose that liver stiffness promotes PDAC liver metastasis through coordinated tumor‐intrinsic mechanosensing and an HSC‐driven paracrine amplification loop, and we identify a Piezo1‐ERS‐GMFG‐TNS4 cascade as a central component of this niche‐centric program (Figure 7).

## 4 Methods

### 4.1 Materials and Cell Lines

GelMA‐60 (60±5% substitution; EFL, Suzhou, China) and photoinitiator LAP were commercially sourced. Healthy male C57BL/6 mice (6–8 weeks) were obtained from the Laboratory Animal Centre of Nantong University and maintained under specific pathogen‐free conditions, with all animal protocols approved by the Institutional Animal Ethics Committee. Cell lines‐including human hepatic stellate cells (LX‐2), pancreatic cancer cells (MIA‐PaCa2, CFPAC‐1), mouse pancreatic cancer cells (Pan02)‐were acquired from ATCC (Shanghai, China). For hydrogel preparation, GelMA solids were dissolved in 0.25% (w/v) LAP solution at 60°C.

### 4.2 Cell Culture

Human LX‐2, CFPAC‐1 and Mouse Pan02 cells were cultured in DMEM medium (AC01L043, Life‐iLab, China) containing 10% fetal bovine serum (FBS, Vazyme, China) respectively, 1% P/S (penicillin and streptomycin, AC03L332, Life‐iLab, China) at 37°C and 5% CO_2_. Human MIA‐PaCa2 cells were additionally supplemented with 1.25% heat‐inactivated horse serum (Gibco, USA) and 1% sodium pyruvate (Gibco, USA) compared to CFPAC‐1 cells. When the cells reached 70%–80% fusion, the adherent cells were digested with 0.25% trypsin‐EDTA (Invigen, USA) and transferred to new petri dish for further expansion. Cells cultured on hydrogels were isolated after incubation for 15 min in medium containing gel lyser (EFL‐GM‐LS‐001, Suzhou, China). Vehicle controls were included in all drug‐treatment experiments. Unless otherwise indicated, LX‐2 cells were cultured on stiffness‐defined GelMA hydrogels for 5 days before collection for immunofluorescence, Western blotting, qRT‐PCR, or Fluo‐4 AM assays. For pharmacological modulation under stiffness conditioning, Yoda1 or GsMTx4 was added during the final 24 h before sample collection. For ER stress intervention experiments, 4‐PBA or TUDCA was applied for 24 h, whereas BAPTA‐AM was added 1 h before harvest.

### 4.3 Conditioned Medium (CM) Preparation and Intra‐Tail Vein Injection

As described previously [53], Pan02 and MIA‐PaCa2 cells were cultured to ≈70%–80% confluence, washed once with PBS, and incubated in basal medium for 24 h to prepare conditioned medium (CM). Basal medium treated in parallel served as the vehicle control. Collect the supernatant, remove cells and cell debris by stepwise centrifugation (300 × g for 5 min, 2000 × g for 10 min), and filter through a 0.22 µm membrane. Subsequently, further concentrate using an ultrafiltration tube (3 kD, Merck Millipore, Germany) to obtain concentrated CM (1 µg µL^−1^). The CM was aliquoted and stored at −80°C for subsequent experiments. For HSC‐derived conditioned medium, LX‐2 cells were cultured on low‐ or high‐stiffness hydrogels for 5 days, washed once with PBS, and then incubated in serum‐free or basal medium for the final 24 h. The collected supernatants were centrifuged to remove cell debris and used as low‐stiffness conditioned medium (L‐CM) or high‐stiffness conditioned medium (H‐CM). The protein concentrations of L‐CM and H‐CM were determined by BCA protein assay (Beyotime, Shanghai). Immunocompetent C57BL/6 mice received Pan02‐CM or solvent via tail vein injection (200 µL per dose) twice weekly for 6 weeks; nude mice received MIA‐PaCa2‐CM or solvent under the same protocol.

### 4.4 Isolation and Characterization of Extracellular Vesicles

Conditioned medium from HSC cultures was collected and subjected to sequential centrifugation to isolate extracellular vesicles (EVs). Briefly, the supernatant was centrifuged at 300 × g for 5 min, 2000 × g for 20 min, and 20 000 × g for 30 min to remove dead cells, cell debris, and larger particles. The clarified supernatant was then ultracentrifuged at 100 000 × g for 70 min to obtain the EV pellet. The pellet was resuspended in PBS and used as the EV fraction, while the supernatant after ultracentrifugation was collected as the EV‐free fraction.

EV morphology was examined by transmission electron microscopy, and particle size distribution was analyzed by NTA. EV marker proteins (CD63 and CD9) and the negative marker Calnexin were detected by Western blotting. GMFG expression in the EV and EV‐free fractions was also analyzed by Western blotting.

### 4.5 Biochemical Indicator Testing

Aspartate aminotransferase (AST) and alanine aminotransferase (ALT) were quantified using commercial assay (BC1555, BC1565) kits from Solarbio (Beijing, China).

### 4.6 Mouse Model of Pancreatic Cancer Liver Metastasis

The construction of high stiffness liver mouse model referred to previously published articles [54, 55]. In brief, 50% CCl_4_ olive oil solution (3 mL kg^−1^) into the abdominal region of 6 weeks old male mice twice in the first week, followed by 10% CCL_4_ (2 mL kg^−1^) intraperitoneally injected twice a week for 5 weeks. GsMT×4 (MCE) was dissolved in PBS at a dose of 0.5 mg kg^−1^ and administered every other day. The control group received intraperitoneal injections of olive oil/PBS. Then 10 days later, mouse pancreatic adenocarcinoma cells (Pan02, 1 × 10^6^) were injected into the splenic capsule of each mouse and remove spleen after 20 min of circulation. Mice euthanized after 2/4 weeks were used for subsequent experiments. This research was approved by the Animal Ethics Committee of Nantong University (Approval No. P20250306‐O43).

### 4.7 Human Pancreatic Cancer Liver Metastasis Sample

Human pancreatic cancer liver metastasis sample was procured from patients under written informed consent. The experimental protocol complied with related legal regulations and was approved by the Institutional Ethics Committee of the Affiliated Hospital of Nantong University (Approval No. 2021‐K136).

### 4.8 Mechanical Strength Measurement

The stiffness of human liver metastasis specimens was measured by a nanoindentation instrument. At the endpoint of tail vein injection (48–72 h after the final injection) or CCL_4_ injection (10 days after the final injection), immediately remove the liver and place it in cold PBS to maintain continuous hydration. Prepare cylindrical tissue cores from standardized liver lobes and trim to uniform thickness; collect two independent cores per mouse (*n* = 6). Quantify liver stiffness using unconfined compression testing on a universal testing machine (C42.503, MTS Systems, USA). After establishing contact with a micro‐preload, apply an axial compressive load vertically downward at 2 mm min^−1^ and calculate the elastic modulus based on the linear range of the stress–strain curve. Hydrogel samples were prepared as 9 mm diameter and 10 mm height cylinders. Axial compression was performed vertically at 5 mm min^−1^ using an electronic universal materials testing machine. Axial compression was performed vertically downward at a rate of 5 mm min^−1^ and the modulus was calculated from the linear range of the stress‐strain curve. GelMA hydrogels were prepared at 4%, 5%, 6%, 7%, 8%, and 9% (w/v), yielding measured Young's moduli of 4.82 ± 0.20, 6.95 ± 0.82, 9.86 ± 0.79, 18.49 ± 0.03, 22.69 ± 0.69, and 29.67 ± 2.28 kPa, respectively. Based on these measurements, 4%, 6%, and 7% GelMA hydrogels were selected as the low‐, medium‐, and high‐stiffness conditions for subsequent experiments.

### 4.9 Live/Dead Staining

Cells were inoculated onto hydrogels spread in confocal dishes for 5 days and stained with a live/dead staining kit (Dojindo, Japan). After 20 min of incubation, images were taken with a confocal microscope (LSM900, Zeiss). Live cells appeared green and dead cells appeared red.

### 4.10 Scanning Electron Microscopy

The prepared cured hydrogel samples were placed in a freeze dryer to dry completely, after which platinum was sprayed on the surface of the samples. The samples were placed into an electron microscope loading stage, and the internal morphology was recorded by scanning electron microscope (SEM) (Hitachi, Japan).

### 4.11 Rheological Characterization

The rheological properties of hydrogels were characterized using a rotational rheometer (Thermo Scientific, Haake RS6000, USA). Measurements were performed at a fixed angular frequency of 5 rad s^−1^ and 1% strain under physiological temperature (37°C). Frequency‐dependent moduli were evaluated at 1 Hz across a strain amplitude range of 0.001% to 10%, with storage modulus (G′) and loss modulus (G″) recorded.

### 4.12 CCK‐8 Assay

To assess the rate of cell proliferation using the CCK‐8 kit (Cell Counting Kit 8 from Dojindo, Japan), MIA‐PaCa2 and CFPAC‐1 cells that had received stimulation were cultured in 96‐well plates at 37°C and 5% CO_2_. Optical density at 450 nm was measured at the indicated time points (0, 24, 48, and 72 h).

### 4.13 Fluo‐4 AM Staining

Cells implanted on hydrogels for 5 days were incubated with 2 µm Fluo‐4 AM solution for 30 min and DAPI (AS21L123, Life‐iLab, China) solution for 15 min at 37°C. Then cells were washed by PBS three times and images were acquired by confocal microscopy.

### 4.14 RNA Isolation and qRT‐PCR

Total RNA was extracted by lysing cells with Trizol reagent (AN51L758, Life‐iLab, China). The cDNA was obtained by reverse transcription using a reverse transcription kit (Thermo Scientific, K1622, USA). Fast SYBR Green qPCR kit (Servicebio, G3323‐05, China) was used to perform qRT‐PCR. Primers were designed and synthesized by Sangon Biotech (Shanghai, China) and all primer sequences are displayed in Table S2. Relative expression of mRNA levels were calculated by the 2^−ΔΔCt^ method.

### 4.15 Phalloidin and Nile Red Staining

Human pancreatic cancer cells implanted on the hydrogel were cultured in confocal dishes for 5 days, fixed in 4% paraformaldehyde for 20 min, and permeabilized with 0.2% Triton X‐100 for 10 min. Then F‐actin was stained with phalloidin solution (CA1610, Solarbio) for 60 min while lipid was stained with Nile Red (HY‐D0718, MCE) for 30 min both at 37°C. Nuclei were stained with DAPI (BL105A, biosharp, China). Confocal microscope (LSM900) was used to obtain images. Image J software (NIH, Bethesda, MD, USA) was utilized for the quantitative analysis.

### 4.16 Immunofluorescence and Immunohistochemistry

The cells were fixed and incubated overnight with primary antibodies at 4°C. Fluorescent secondary antibody was incubated for 2 h and DAPI (AS21L123, Life‐iLab, China) was incubated for 20 min at room temperature. Imaging was recorded by confocal microscope (LSM900, Zeiss, Germany). Tissue sections for Immunofluorescence (IF) and Immunohistochemistry (IHC) were dewaxed and rehydrated in xylene and gradient ethanol, respectively. They were then incubated with primary antibody at 4°C overnight. The sections were then washed three times with PBS and incubated with the secondary antibody for 1 h at room temperature, then the microscope acquires the image. The detailed information of antibodies for IF and IHC can be found in Table S1. Image J software (NIH, Bethesda, MD, USA) was utilized for the quantitative analysis. For immunofluorescence and immunohistochemistry quantification, 3–5 randomly selected fields were analyzed per sample and averaged as one biological replicate.

### 4.17 Western Blot Assay

Total protein extraction utilized RIPA lysis buffer (AP01L014, Life‐iLab, China) and protease and phosphatase inhibitors (AP02L192, Life‐iLab, China) mixture, with concentrations quantified by BCA assay (Cat.PA002‐01A, Novoprotein, Shanghai, China). Equal protein aliquots underwent SDS‐PAGE (SK621075, Coolaber, China) separation and subsequent transfer to PVDF membranes. After blocking with 5% skim milk, membranes were incubated with primary antibodies (4°C, overnight) followed by species‐matched secondary antibodies (room temperature, 2 h). After incubation with ECL chemiluminescent buffer (AP34L065, Life‐iLab, China), the protein bands were visualized using a Tanon imaging system (Shanghai). Antibody specifications were provided in Table S1.

### 4.18 Plasmids, siRNAs, Lentiviral Vectors and Cell Transfections

The Flag‐tagged GMFG overexpression plasmid and HA‐tagged TNS4 overexpression plasmid were synthesized by Genepharma (Suzhou, China). siRNA sequences targeting Piezo1, GMFG and TNS4 were designed and synthesized by Tsingke (Nanjing, China), with detailed sequences provided in Table S3. Cells were analyzed 48 h after plasmid or siRNA transfection unless otherwise indicated. Cell transfection was performed using jetPRIME transfection reagent (Polyplus, Strasbourg, France) according to the manufacturer's protocol. To provide a more precise downstream in vivo intervention, lentiviral vectors encoding shTns4 or non‐targeting control (shNC) were used to establish stable Pan02 cell lines. After infection in the presence of polybrene, cells were selected with puromycin for stable integration. Knockdown efficiency was confirmed by Western blotting before in vivo use.

### 4.19 Co‐Immunoprecipitation (Co‐IP)

The supernatant after cell lysis was cultured overnight with control IgG or specific antibodies. Subsequently, protein A/G agarose beads (Bioworld Technology, Louis Park, USA) were introduced and the mixture was incubated at 4°C for 2 h. After washing the protein antibody complex obtained in PBS, discard the supernatant, and use sodium dodecyl sulfate polyacrylamide gel electrophoresis to analyze the retained samples.

### 4.20 GST Pull‐Down Assay and Fluorescence Uptake/Co‐Localization Analysis

To examine whether GMFG can directly associate with TNS4, GST pull‐down assays were performed using recombinant GST‐tagged GMFG protein, referencing published article [56]. Briefly, GST‐GMFG plasmids were transformed into E. coli, and protein expression was induced with IPTG. The purified GST protein was purchased from Sino Biological, China (Cat. G52‐30U). Bacterial lysates were incubated with glutathione agarose beads to purify GST‐GMFG fusion proteins. Purified bait proteins immobilized on beads were then incubated with lysates from pancreatic cancer cells expressing endogenous or exogenous TNS4. After extensive washing, bound proteins were eluted and analyzed by SDS‐PAGE followed by Coomassie staining (AP11L124, Life‐iLab, China) and Western blotting with anti‐TNS4 antibody.

For fluorescence uptake/co‐localization analysis, recombinant GMFG protein was labeled with a red fluorescent protein‐labeling kit according to the manufacturer's instructions, and excess free dye was removed before use. Pancreatic cancer cells were incubated with labeled GMFG for the indicated time periods, fixed with 4% paraformaldehyde (AC28L112, Life‐iLab, China), permeabilized with 0.1% Triton X‐100 (Macklin, Shanghai), and incubated with anti‐TNS4 primary antibody followed by the corresponding fluorescent secondary antibody. Nuclei were counterstained with DAPI. Images were acquired using a confocal fluorescence microscope, and representative fields were selected to assess intracellular punctate distribution of exogenous GMFG and its spatial overlap with TNS4.

### 4.21 Enzyme‐ Linked Immunosorbent Assay

After the indicated treatment, cells were incubated in serum‐free medium for the final 24 h, and conditioned medium was collected and centrifuged at 1000 rpm for 5 min at 4°C. According to the manufacturer's instructions, enzyme‐linked immunosorbent assay (ELISA) kits were used to measure GMFG in HSC‐derived conditioned medium and triglycerides (TG) in cell lysates or supernatants as indicated.

### 4.22 Wound‐Healing Assay

Pancreatic cancer cells were treated with conditioned medium (CM) for 48 h. The adherent cells were scraped using a 10 µL pipette tip. Floating cells were washed using PBS and the remaining cells were cultured in serum‐free medium. Images were recorded with a microscope at different time points.

### 4.23 Transwell Assay

During assay, complete medium (500 µL) was filled into a 24‐well plate (Cat.725321, NEST Biotechnology, China). Subsequently, transwell chambers were inserted into each well and the prepared cell suspension was added to each chamber. After 48 h of incubation, cells were fixed, stained and washed. Randomly selected fields of view were imaged and recorded using a microscope.

### 4.24 Transmission Electron Microscopy

The prepared cell samples were fixed with glutaraldehyde at 4°C overnight. Samples were rinsed with 0.1 M phosphoric acid and then fixed and rinsed again. The samples were then subjected to gradient dehydration and permeabilization with ethanol and acetone. Finally, the embedded samples were sectioned and double‐stained with uranium‐lead, and images were acquired using transmission electron microscopy (TEM).

### 4.25 Cell Colony Formation

Pancreatic cancer cells stimulated by specific processing were collected and washed, and then the cell suspension was cultured with a density of about 800–1000 cells per well and cultured for 10–14 days. When cell clusters visible to the naked eye appear in the six well plate, rinse the cells with PBS for 2–3 times. Then the cells were fixed, stained, washed and captured under a camera to record the formation of colonies.

### 4.26 EdU

Cell proliferation assays were performed by a cell proliferation kit with EdU (C0081S, Beyotime, Shanghai, China). Briefly, pancreatic cancer cells that stimulated by specific processing were incubated with 10 µM 5‐ethynyl‐2'‐deoxyuridine (EdU) for 2 h at 37°C, fixed with paraformaldehyde for 20 min, then treated with 0.2% TritonX‐100 for 10 min, and rinsed three times with PBS. Click reaction mixture (100 µL) was added and the mixture was incubated for 30 min, followed by Hoechst 33342 (C0081S, Beyotime) for 20 min. Images were captured with a confocal microscope (LSM900).

### 4.27 Pancreatic Cancer Cells Adhesion Assay

Pancreatic cancer cells (CFPAC‐1 and MIA‐PaCa2) were pretreated with the indicated conditioned media or recombinant GMFG for 48 h, labeled with calcein AM (2 µL mL^−1^, Solarbio, IC4630, China) at 37°C for 30 min, and then resuspended and added to the hydrogel culture system containing hepatic stellate cells. After co‐incubation for 2 h, the non‐adherent pancreatic cancer cells were removed, and the calcein AM labeled pancreatic cancer cells were imaged with a confocal microscope (Zeiss, LSM 900, Germany).

### 4.28 Hematoxylin and Eosin (HE) and Masson Staining

Tissue sections were deparaffinized and rehydrated. Nuclei were stained with hematoxylin, differentiated in acid ethanol and blued in ammonia water, followed by cytoplasmic counterstaining with eosin (AS11L011, Life‐iLab, China). For Masson staining, sections were fixed in Bouin solution (60°C, 1 h), stained sequentially with Weigert hematoxylin, Biebrich scarlet‐acid fuchsin, and aniline blue, then treated with phosphomolybdic acid for collagen differentiation. Finally, sections were dehydrated, cleared in xylene, and mounted with neutral balsam.

### 4.29 Transcriptomics Sequencing

The mRNA sequencing of LX‐2 cells was done by Illumina platform (OBIO, Shanghai, China) with three replicates per group. Significantly highly expressed genes (log_2_ FC >1, *p* < 0.05) are indicated in red, while significantly low expressed genes (log_2_ FC ←1, *p* < 0.05) are indicated in green. Protein‐protein interactions (PPI) were visualized via STRING database export. The full gene list was further analyzed functionally by the Gene Set Enrichment Analysis (GSEA) Java application.

### 4.30 Endoplasmic Reticulum Tracker (ER‐tracker) and Rhod‐2 AM Staining

After washing prepared cells twice with PBS, incubate them with ER‐Tracker Green (1:1000 dilution; Thermo Fisher, E34251) and Rhod‐2 AM (0.1%; MKBio, MX4507) in pre‐warmed complete medium at 37°C for 1 h under light‐protected conditions, followed by two additional PBS washes; subsequently stain nuclei with Hoechst 33342 in PBS for 20 min at room temperature in the dark, concluding with two final PBS rinses prior to imaging. Images were captured with a confocal microscope (LSM900).

### 4.31 Mass Spectrometry

Samples were detected with Fast Silver Staining Kit (P0017S, Beyotime), and measured on the SDS‐PAGE gels according to the manufacturer's instructions. LCMS/MS analysis was performed on specific bands. Then, protein identification was searched in the National Center for Biotechnology Information (NCBI) database. The simulation docking of binding sites was performed using GRAMM. The protein sequence was searched in UniProtKB, and the sequence was input into SWIEE‐MODEL to search for the optimal protein structure, the output PDB file was used for protein‐protein docking. The combination method of ranking first was selected.

### 4.32 Bioinformatics Analysis

Transcriptome sequencing data underwent Gene Set Enrichment Analysis (GSEA) and Gene Ontology (GO) enrichment using cluster Profiler. We downloaded PDAC liver metastasis tissue of Visium spatial transcriptomics (ST) from GSE281288. To compare the expression of GMFG, we aggregated the counts of all spatialspots to generate a pseudo‐bulk for ST sample, normalizing the data to log2(TPM+1). The expression level of GMFG and fatty acide metabolism score in ST samples was performed and visualized using the R package SpaCET [57]. The “Seurat” R package was used to process the scRNA‐seq data [58]. Cells with fewer than 500 or more than 7500 detected genes, or with mitochondrial gene content exceeding 20%, were excluded from downstream analysis. The data were normalized using a global‐scaling normalization method with a scale factor of 10 000. Highly variable genes were identified using the “vst” method. The data were then scaled and subjected to principal component analysis (PCA) using the Scale Data and RunPCA functions in the Seurat package. To correct for potential batch effects, the Harmony algorithm was applied to the PCA embeddings [59]. Clustering was performed using the FindClusters function with a resolution parameter set to 0.2. The top 15 principal components were used for dimensionality reduction via Uniform Manifold Approximation and Projection (UMAP). Finally, cell clusters were annotated based on canonical marker gene expression profiles.

### 4.33 Statistical Analysis

Statistical Analyses Were Primarily Performed Using GraphPad Prism 8.0 (GraphPad Software, California, USA) and SPSS 28.0 (IBM, USA). In the experimental manipulations, t‐test was used to compare data between two groups, while one‐way ANOVA was used to compare more data over two groups. A *p*‐value < 0.05 was considered statistically significant.

## Author Contributions

**B.Z**., **J.W**., and **X.Z**. contributed equally to this work. **B.Z**., **Q.G**., **Y.G**., and **Y.L**. conceived and designed the project. **B.Z**., **J.W**., and **X.Z**. performed the experiments. **B.Z**., **J.W**., and **X.Z**. analyzed the sequencing data. **J.Y**., **X.C**., **X.G**., **Q.R**., **T.Y**., and **D.W**. helped with the experiments. **B.Z**., **J.W**., and **X.Z**. analyzed the data. **B.Z**. and **Y.L**. provided grants. **B.Z**., **J.W**., **X.Z**., **J.Y**., **X.C**., **Y.G**., and **Y.L**. wrote and revised the manuscript.

## Conflicts of Interest

The authors declare no conflicts of interest.

## Supporting information




**Supporting File**: advs76173‐sup‐0001‐SuppMat.docx.

## Data Availability

The data that support the findings of this study are available from the corresponding author upon reasonable request.
