# Supplementary material for: Stiffness‐Activated Stellate Cells Drive Pancreatic Cancer Liver Colonization via GMFG‐TNS4 Signaling
Source: Adv Sci (Weinh). 2026 Jun 18:e76173. Online ahead of print. doi: 10.1002/advs.76173 (PMC13337057; doi:10.1002/advs.76173)
Supplement: Supplementary file 1 — Supporting File: advs76173‐sup‐0001‐SuppMat.docx. [file ADVS-9999-e76173-s001.docx]

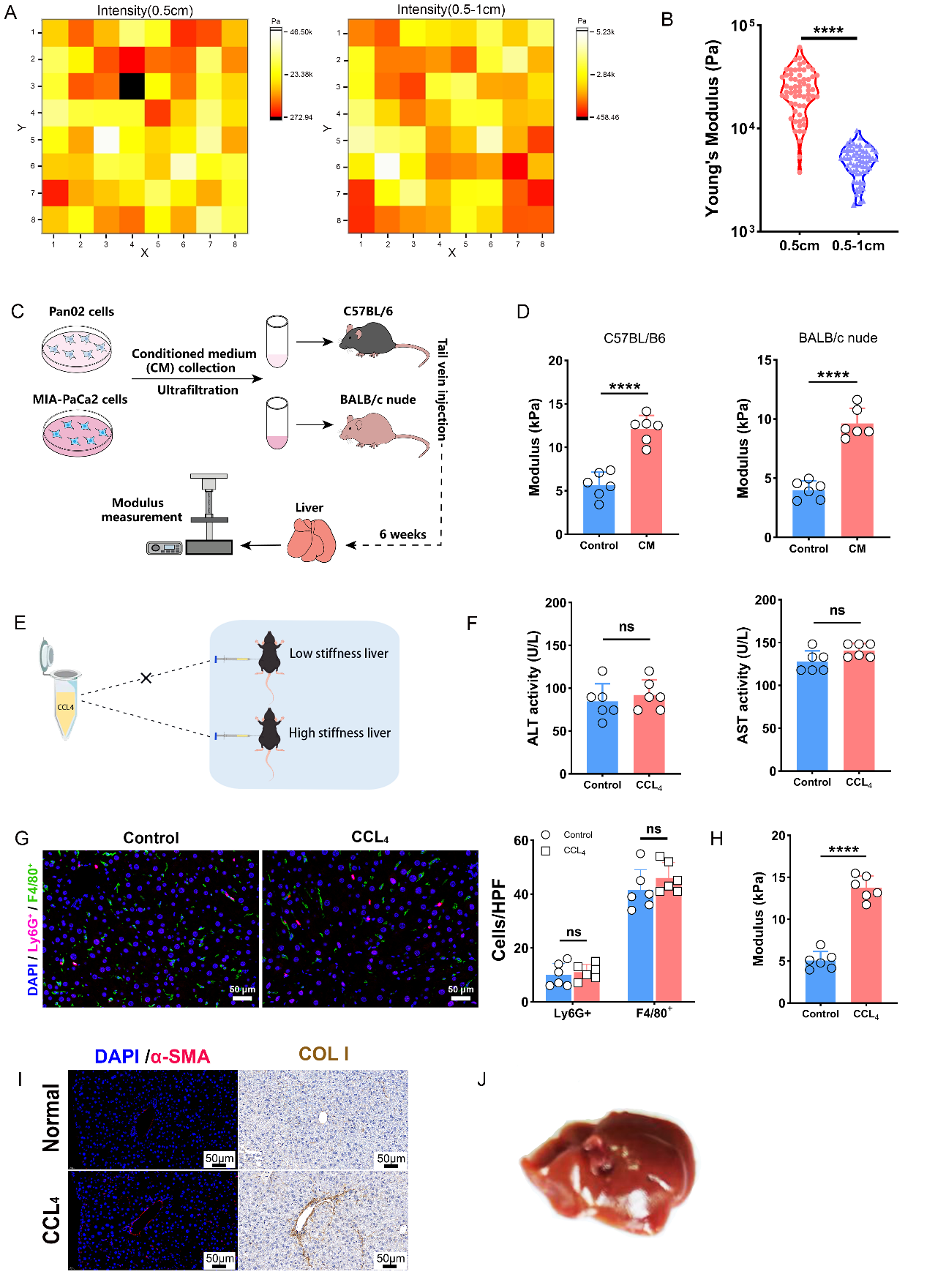


**Figure. S1. Clinical and pre-metastatic validation of liver stiffening and characterization of the fibrotic liver model.** (A, B) Nanoscale indentation measurements and quantification of liver tissue stiffness at different distances from human liver metastatic lesions. (C, D) Schematic of tail-vein administration of pancreatic cancer cell–derived conditioned media and corresponding liver stiffness measurements in mice (n=6). (E) Schematic of CCl₄-induced high-stiffness liver modeling. (F) Serum alanine aminotransferase (ALT) and aspartate aminotransferase (AST) levels measured prior to tumor inoculation after recovery (n=6). (G) Immunofluorescence staining and quantification of Ly6G⁺ neutrophils and F4/80⁺ macrophages showing comparable inflammatory infiltration between groups (n=6). (H) Liver stiffness measurement following recovery and prior to tumor inoculation (n=6). (I) Representative α-SMA immunofluorescence and collagen I IHC in livers prior to tumor inoculation. (J) Control experiment showing no spontaneous tumor formation in the CCl₄-only group. Scale bars are indicated in the images. All data are presented as mean ± SD. Statistical analysis was performed using two-tailed Student’s *t*-test (two groups) or one-way ANOVA. *P<0.05, **P<0.01, ***P<0.001, ****P<0.0001.


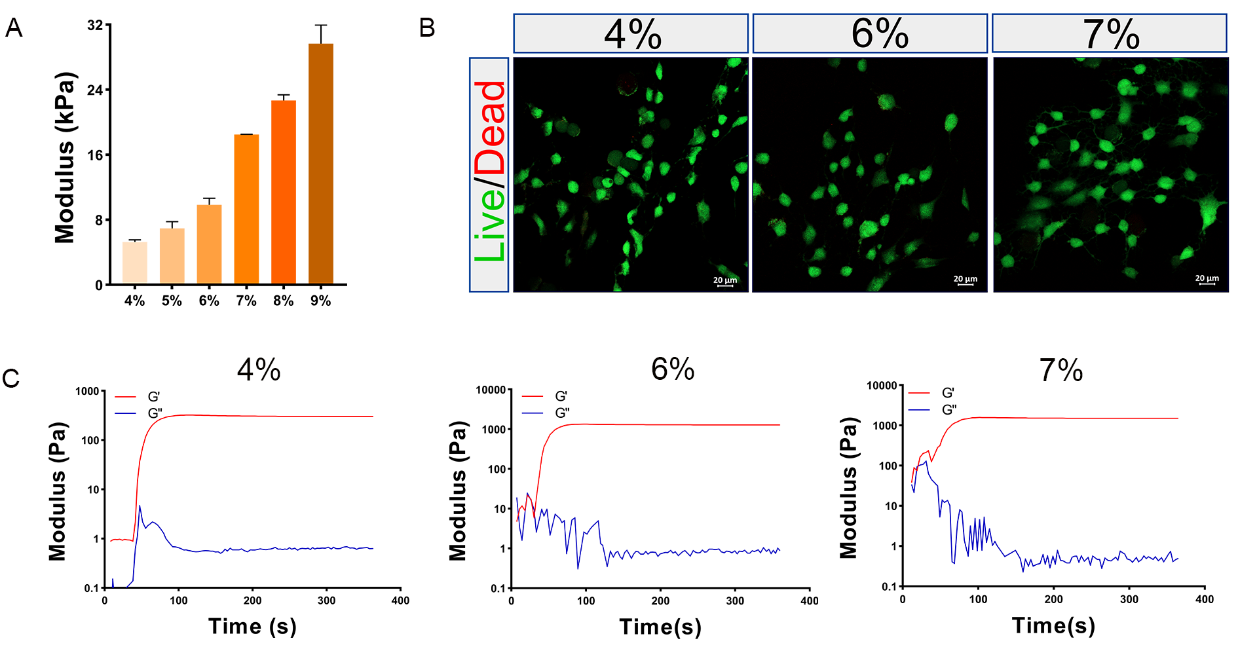


**Figure. S2. Selection and characterization of GelMA hydrogels matched to liver stiffness ranges.** (A) Workflow for screening hydrogels. GelMA hydrogels at 4%, 5%, 6%, 7%, 8%, and 9% (w/v) showed measured Young’s moduli of 4.82 ± 0.20, 6.95 ± 0.82, 9.86 ± 0.79, 18.49 ± 0.03, 22.69 ± 0.69, and 29.67 ± 2.28 kPa, respectively (n=3). (B) Live/dead staining assessing biocompatibility of low/medium/high stiffness GelMA hydrogels. (C) Rheological characterization showing stable gelation after UV crosslinking. Scale bars are indicated in the images. All data are presented as mean ± SD.


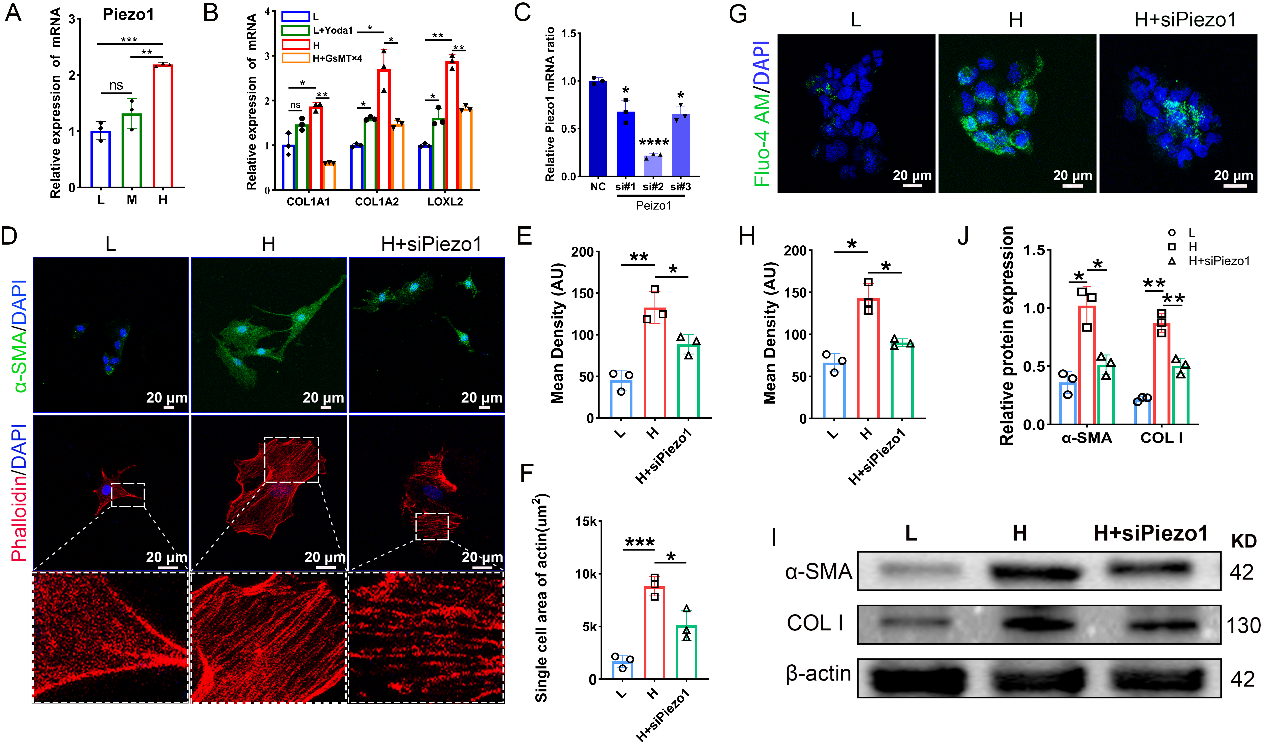


**Figure. S3. Genetic suppression of Piezo1 attenuates stiffness-induced HSC activation and Ca²⁺ influx.** (A) Relative PIEZO1 mRNA expression in HSCs cultured on low/medium/high stiffness hydrogels (n=3). (B) Effects of Yoda1 and GsMTx4 on COL1A1, COL1A2, and LOXL2 mRNA expression in stiffness-conditioned HSCs (n=3). (C) Validation of Piezo1 knockdown efficiency by siRNA (n=3). (D–F) Representative α-SMA and phalloidin staining with quantification showing reduced HSC activation and spreading after Piezo1 knockdown under high stiffness (n=3). (G, H) Fluo-4 AM imaging and quantification showing diminished stiffness-associated Ca²⁺ influx after Piezo1 knockdown (n=3). (I, J) Western blotting and quantification showing reduced α-SMA and collagen I expression following Piezo1 knockdown (n=3). Scale bars are indicated in the images. All data are presented as mean ± SD. Statistical analysis was performed using two-tailed Student’s *t*-test (two groups) or one-way ANOVA. *P<0.05, **P<0.01, ***P<0.001, ****P<0.0001.


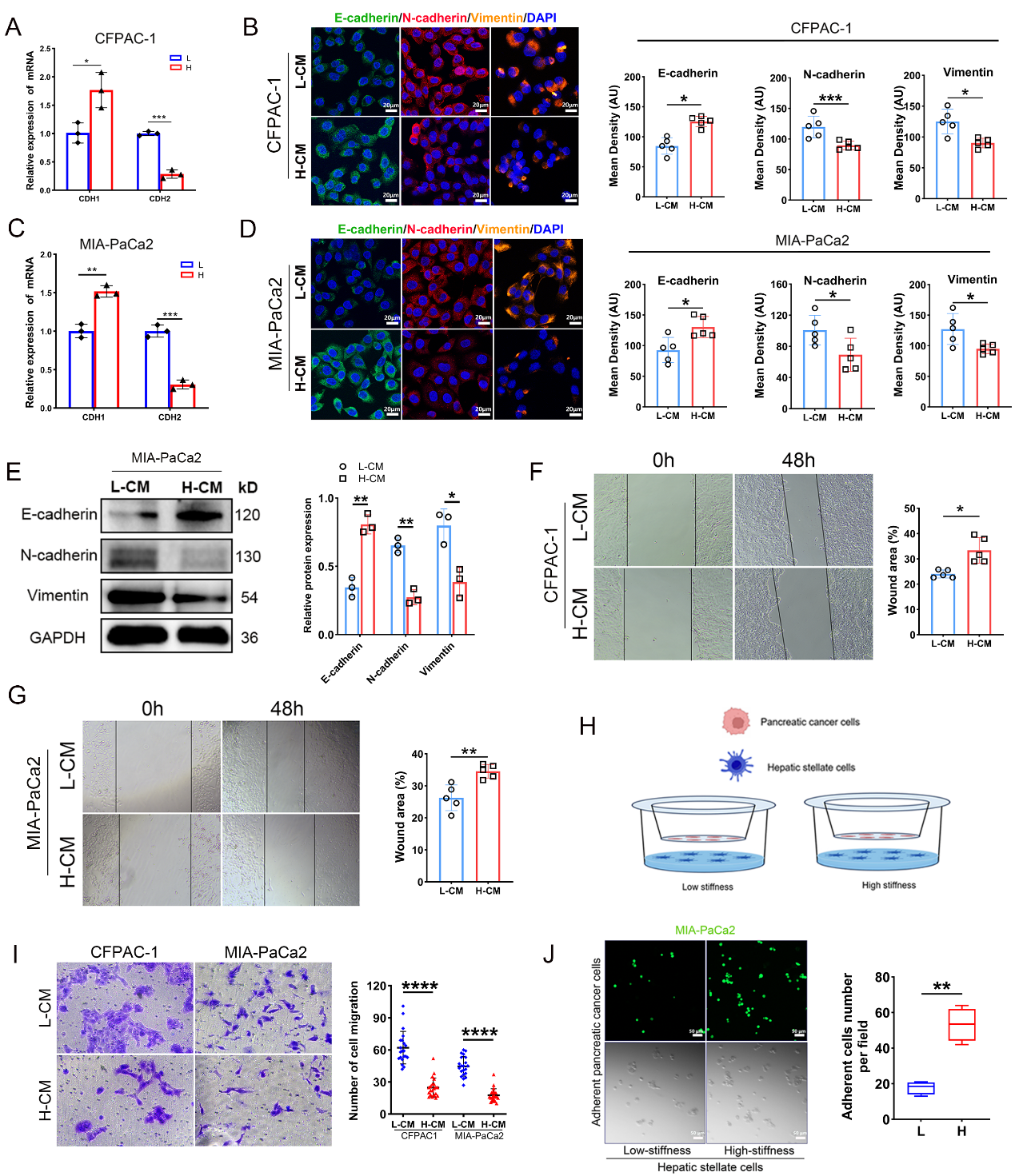


**Figure. S4. H-CM promotes epithelial features and suppresses motility programs in pancreatic cancer cells.** (A–E) Quantitative PCR (n=3), immunofluorescence (n=5), and Western blotting analyses (n=3) showing increased CDH1/E-cadherin and decreased CDH2/N-cadherin/vimentin in CFPAC-1 and MIA-PaCa2 cells treated with H-CM versus L-CM. (F–I) Wound-healing and transwell assays showing reduced migratory capacity after H-CM treatment (n=3). (J) Adhesion assay showing altered adhesive behavior after H-CM treatment (n=4). Scale bars are indicated in the images. All data are presented as mean ± SD. Statistical analysis was performed using two-tailed Student’s t-test (two groups) or one-way ANOVA. *P<0.05, **P<0.01, ***P<0.001, ****P<0.0001.


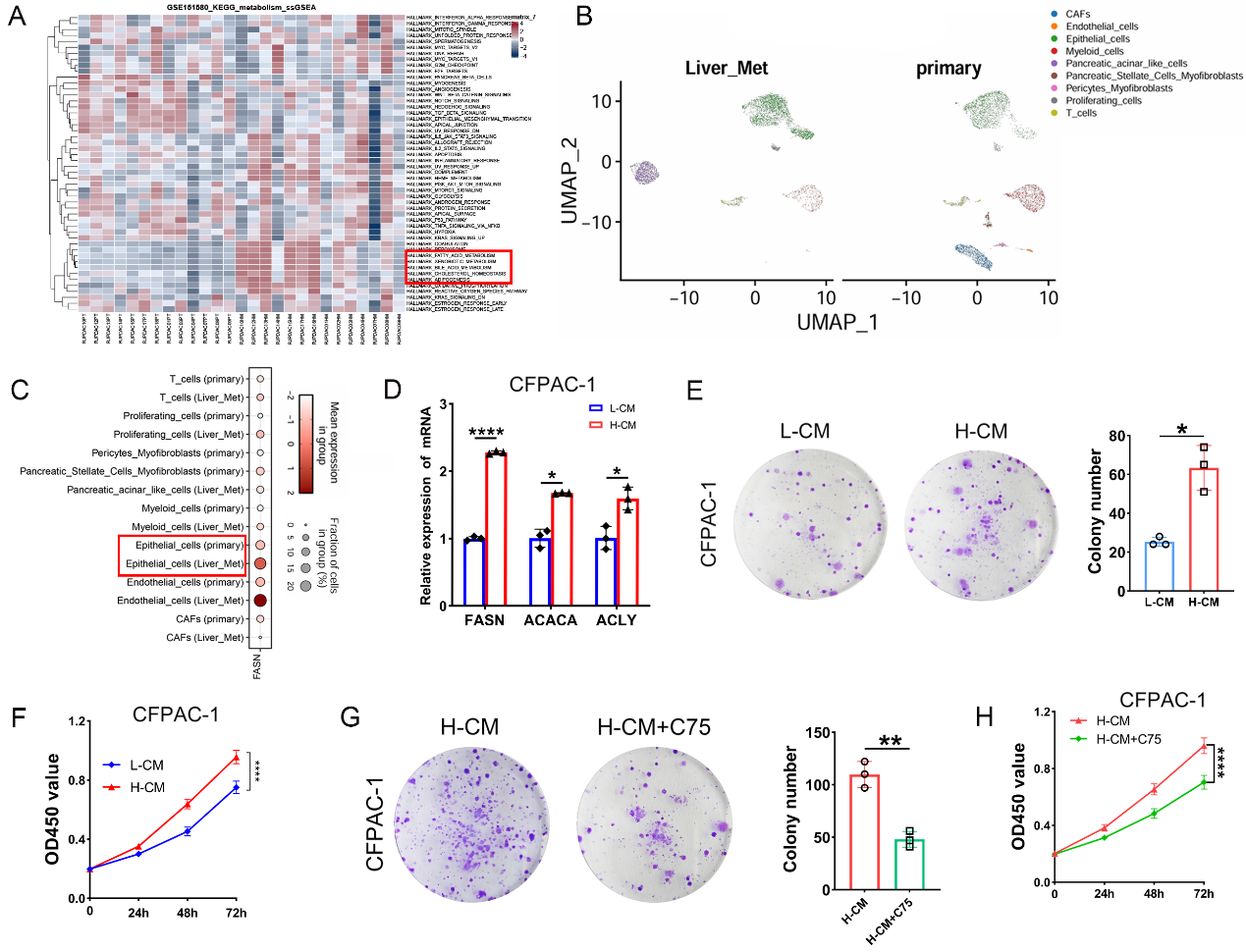


**Figure. S5. Single-cell evidence for lipogenic programs in liver metastasis and validation of H-CM-induced proliferation via FASN.** (A) Single-sample GSEA (ssGSEA) analysis (dataset GSE151580). (B) UMAP visualization of cell populations. (C) Feature plot showing elevated FASN expression in liver metastases compared with primary tumors. (D) Quantitative PCR showing increased FASN/ACC1/ACLY expression in CFPAC-1 cells after H-CM treatment (n=3). (E-H) Colony formation (n=3) and CCK-8 assays (n=8) showing enhanced proliferation after H-CM treatment, which is attenuated by FASN inhibition. Scale bars are indicated in the images. All data are presented as mean ± SD. Statistical analysis was performed using two-tailed Student’s t-test (two groups) or one-way ANOVA. *P<0.05, **P<0.01, ****P<0.0001.


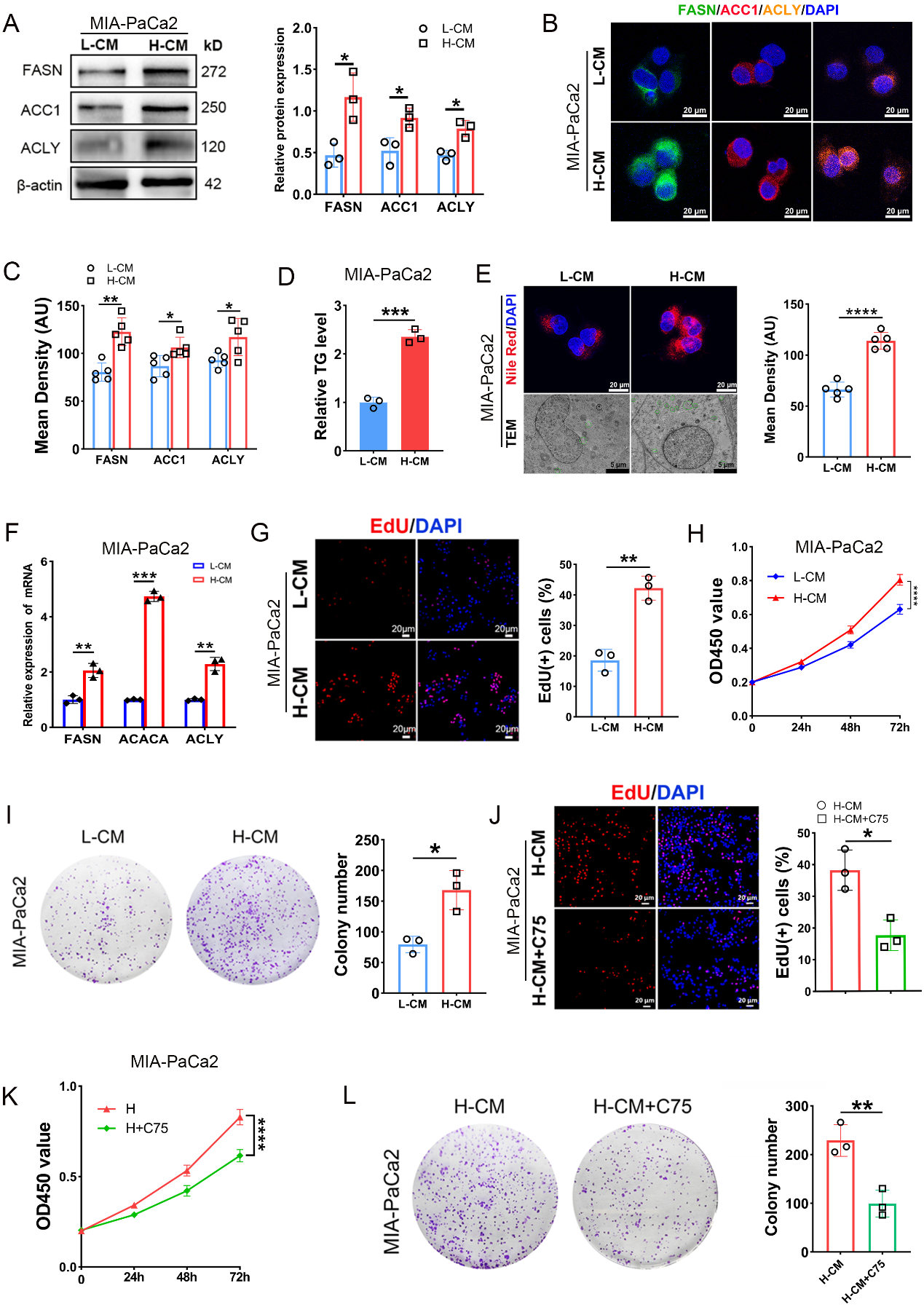


**Figure. S6. H-CM enhances lipid synthesis and proliferation in MIA-PaCa2 cells.** (A-C) Western blotting and immunofluorescence analyses showing increased FASN/ACC1/ACLY in MIA-PaCa2 cells treated with H-CM (n=3). (D) Triglyceride quantification after H-CM treatment (n=3). (E) Nile Red staining (n=5), quantitative analysis, and TEM images showing increased lipid droplet accumulation after H-CM treatment. (F) Quantitative PCR of lipogenic genes after H-CM treatment (n=3). (G-I) EdU (n=3), CCK-8 (n=8), and colony formation (n=3) assays showing increased proliferation after H-CM treatment; C75 suppresses these effects. Scale bars are indicated in the images. All data are presented as mean ± SD. Statistical analysis was performed using two-tailed Student’s t-test (two groups) or one-way ANOVA. *P<0.05, **P<0.01, ***P<0.001, ****P<0.0001.


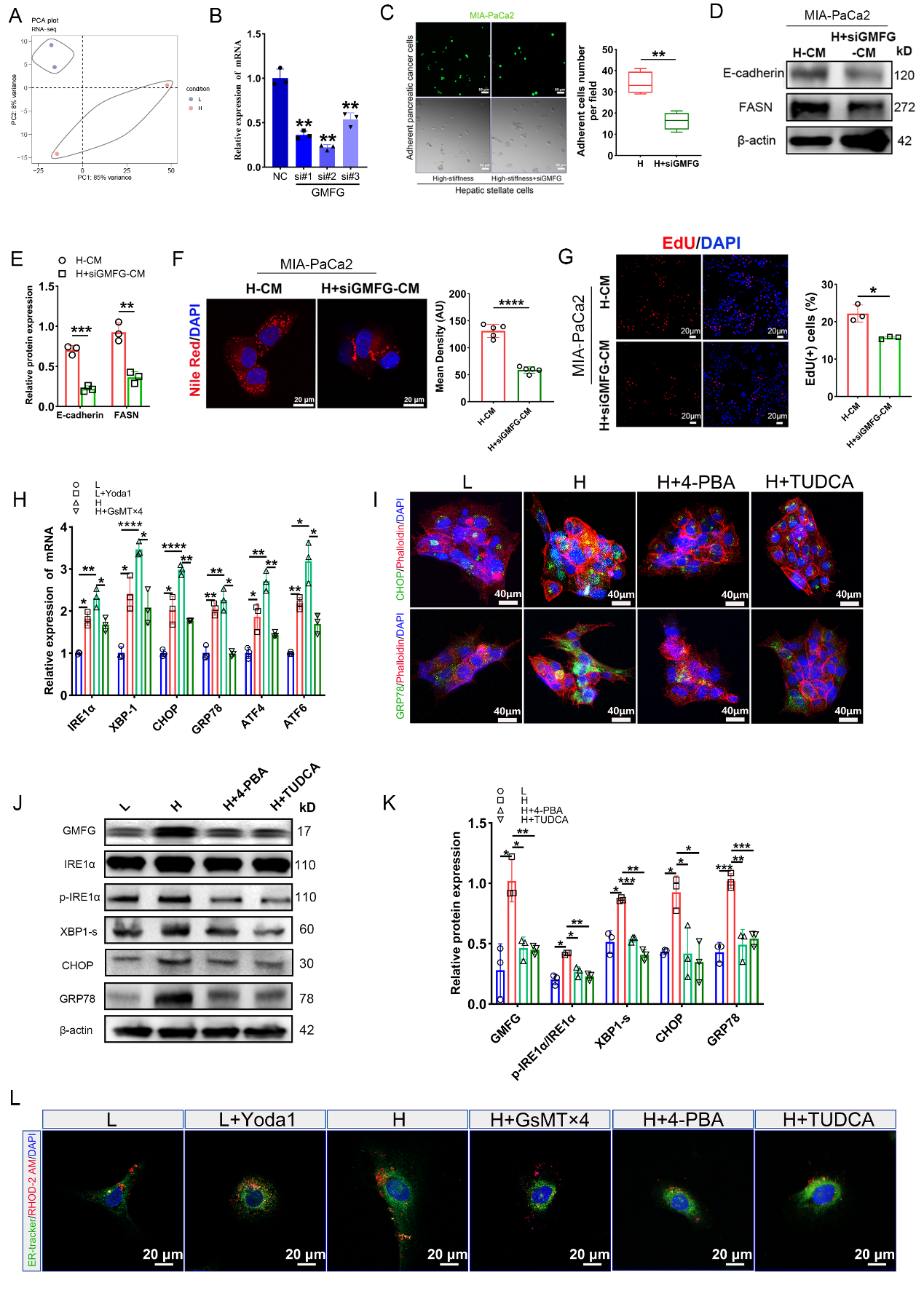


**Figure. S7. Transcriptomic quality control and additional validation linking ER stress to GMFG secretion.** (A) Principal component analysis (PCA) plot of RNA-seq samples. (B) Validation of GMFG knockdown efficiency in HSCs (n=3). (C–G) Functional assays showing reduced cancer cell adhesion (n=4), decreased E-cadherin/FASN expression (n=3), diminished lipid droplet accumulation (n=5), and suppressed proliferation (n=3) after treatment with GMFG-deficient H-CM. (H) ER stress–related gene expression changes after Yoda1 and GsMTx4 treatment (n=3). (I) Western blotting and quantification of ER stress markers following 4-PBA and TUDCA treatment (n=3). (J) Immunofluorescence staining of CHOP and GRP78 with quantification. (K) Quantitative PCR of ER stress–associated genes (n=3). (L) Organelle probe-based imaging tracking Ca²⁺ distribution between endoplasmic reticulum and mitochondria. Scale bars are indicated in the images. All data are presented as mean ± SD. Statistical analysis was performed using two-tailed Student’s t-test (two groups) or one-way ANOVA. *P<0.05, **P<0.01, ***P<0.001, ****P<0.0001.


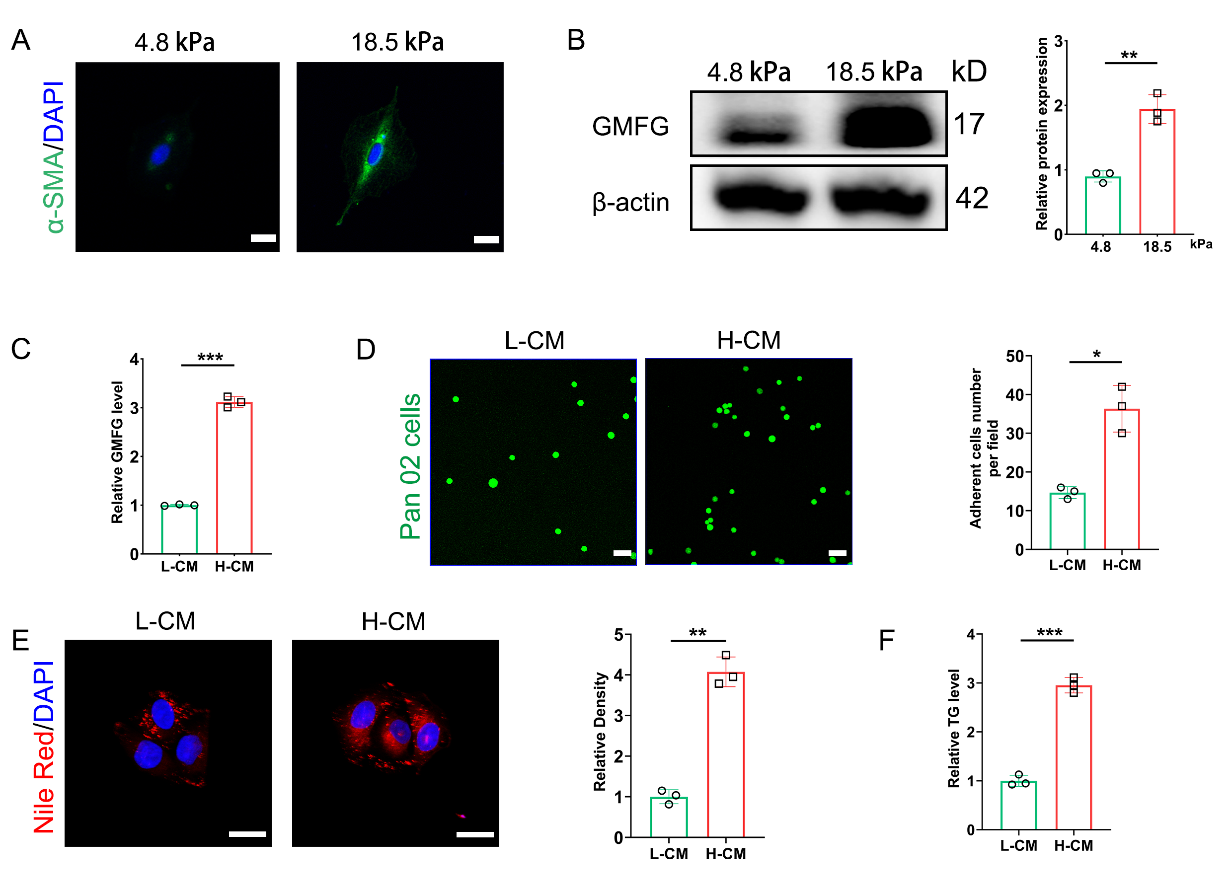


**Figure S8. Primary murine HSCs recapitulate stiffness-induced GMFG synthesis/release and transmit pro-adhesive and pro-lipogenic effects to pancreatic cancer cells.** (A) Representative immunofluorescence images of α-SMA in primary murine hepatic stellate cells cultured on 4.8 kPa or 18.5 kPa substrates. Scale bars is 20μm. (B) Western blot analysis and quantification of GMFG protein expression in primary murine HSCs cultured on 4.8 kPa or 18.5 kPa substrates, with β-actin as loading control. (C) ELISA quantification of GMFG in conditioned media collected from primary murine HSCs cultured on low- or high-stiffness substrates (L-CM and H-CM, respectively). (D) Representative adhesion assay images and quantification of Pan02 cells treated with L-CM or H-CM derived from primary murine HSCs. Scale bars is 50μm. (E) Representative Nile Red staining and quantification of lipid droplet accumulation in Pan02 cells treated with L-CM or H-CM derived from primary murine HSCs. Scale bars is 20μm. (F) Quantification of intracellular triglyceride (TG) levels in Pan02 cells after treatment with L-CM or H-CM derived from primary murine HSCs. All data are presented as mean ± SD. Statistical analysis was performed using two-tailed Student’s t-test. *P<0.05, **P<0.01, ***P<0.001.


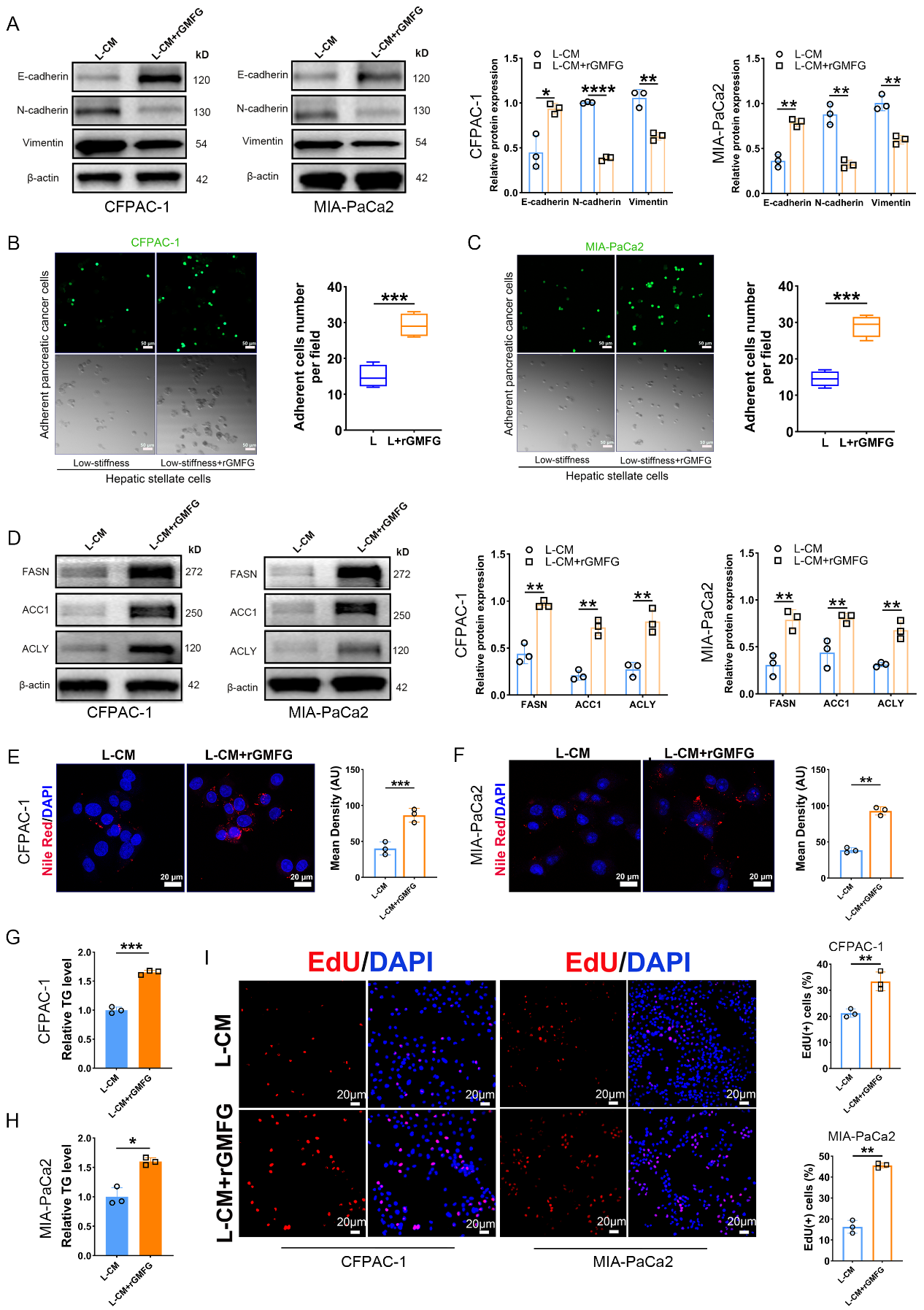


**Figure. S9. Recombinant GMFG supplementation partially recapitulates H-CM induced phenotypes in pancreatic cancer cells.** (A) Western blotting showing that adding recombinant human GMFG (rGMFG) to L-CM modulates epithelial/mesenchymal markers in CFPAC-1 and MIA-PaCa2 cells (n=3). (B, C) Adhesion-related assays with quantification following rGMFG supplementation (n=4). (D) Western blotting showing regulation of lipogenic enzymes (FASN/ACC1/ACLY) by rGMFG supplementation (n=3). (E, F) Nile Red staining and quantification of lipid droplets following rGMFG supplementation (n=5). (G, H) Triglyceride quantification following rGMFG supplementation (n=3). (I) Proliferation assays following rGMFG supplementation (n=3). Scale bars are indicated in the images. All data are presented as mean ± SD. Statistical analysis was performed using two-tailed Student’s t-test (two groups) or one-way ANOVA. *P<0.05, **P<0.01, ***P<0.001, ****P<0.0001.


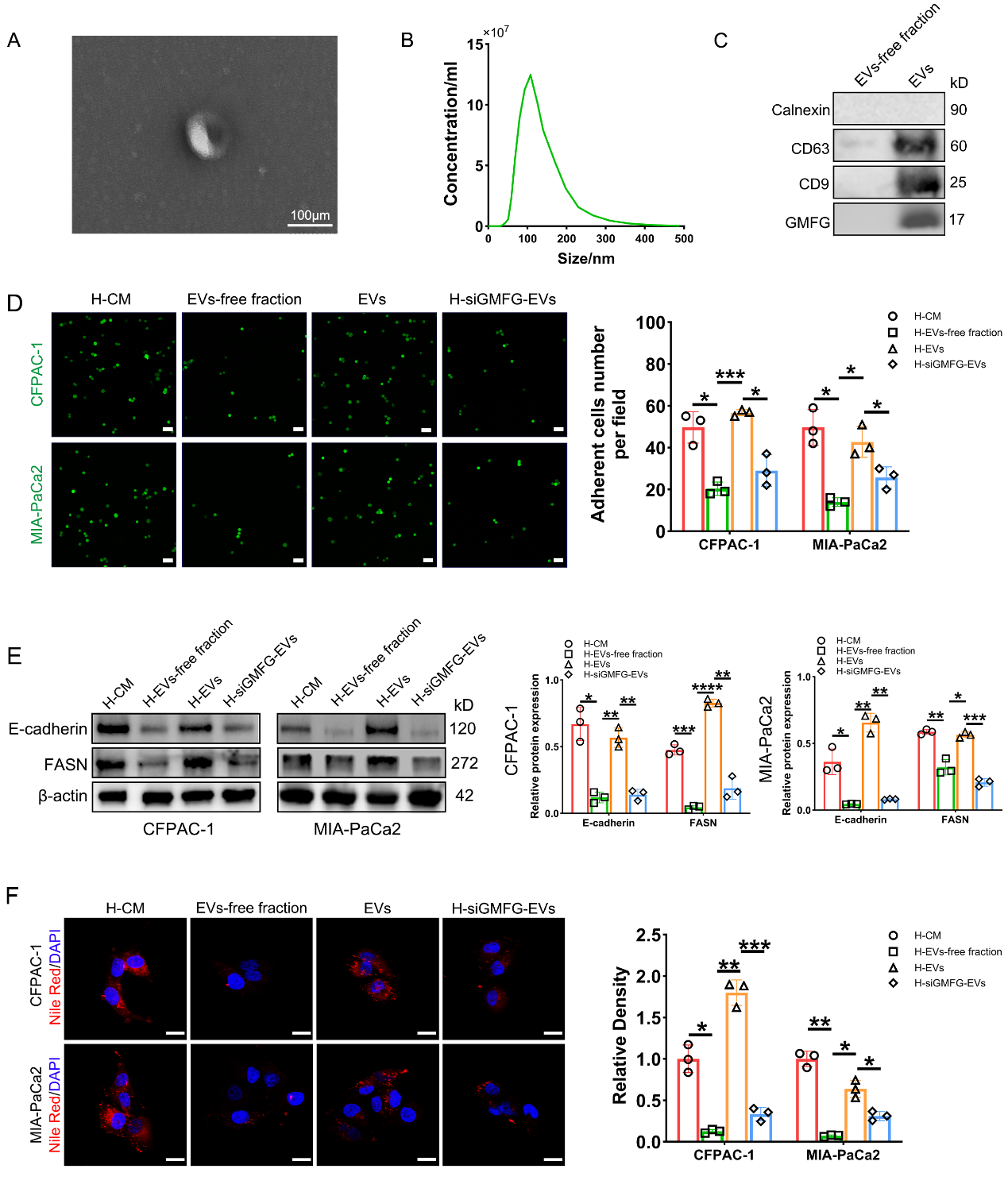


**Figure. S10. GMFG is enriched in HSC-derived extracellular vesicles, and EV-associated GMFG mediates pro-adhesive and pro-lipogenic signaling in pancreatic cancer cells.** (A) Representative transmission electron microscopy image of extracellular vesicles isolated from H-CM. (B) Nanoparticle tracking analysis showing the size distribution of isolated EVs. (C) Western blot characterization of EV-free fraction and EV fraction using the negative marker Calnexin and the canonical EV markers CD63 and CD9. (D) Representative adhesion assay images and quantification in CFPAC-1 and MIA-PaCa2 cells treated with H-CM, H-EV-free fraction, H-EVs, or H-siGMFG-EVs. Scale bars is 50μm. (E) Western blot analysis of E-cadherin and FASN in CFPAC-1 and MIA-PaCa2 cells treated with the indicated fractions, with β-actin as loading control. H-EVs largely phenocopied H-CM in regulating epithelial/metabolic markers. (F) Representative Nile Red staining and quantitative analysis of lipid droplet accumulation in CFPAC-1 and MIA-PaCa2 cells treated with H-CM, H-EV-free fraction, H-EVs, or H-siGMFG-EVs. Scale bars is 20μm. All data are presented as mean ± SD. Statistical analysis was performed using two-tailed Student’s t-test or one-way ANOVA. *P<0.05, **P<0.01, ***P<0.001, ****P<0.0001.


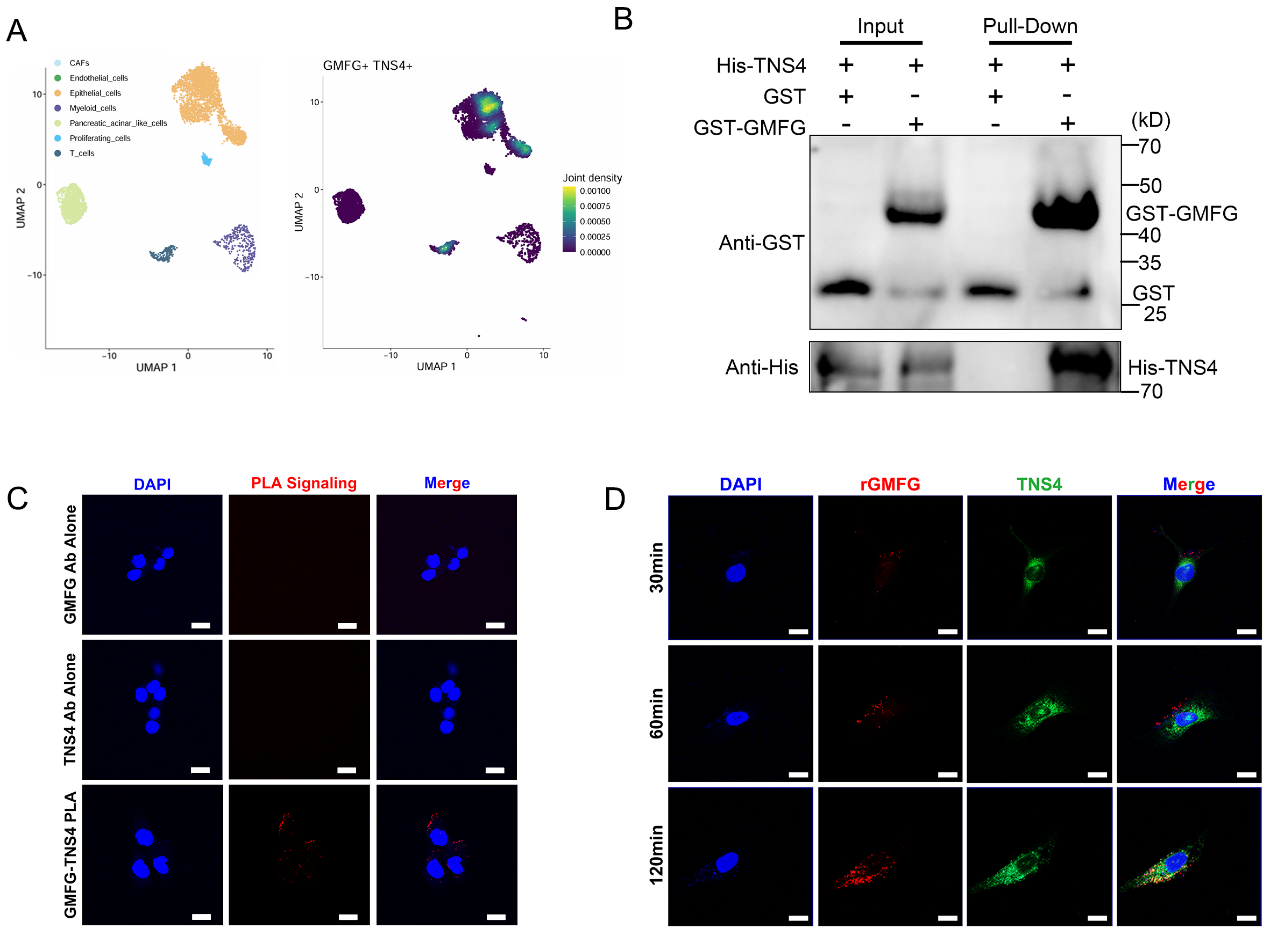


**Figure. S11. Direct interaction, cellular proximity, and uptake-associated localization of GMFG relative to TNS4 in pancreatic cancer cells.** (A) Single-cell analysis of dataset GSE154778 showing that GMFG/TNS4 double-positive cells are enriched within epithelial tumor-cell populations. (B) GST pull-down assay showing direct association between recombinant GST-GMFG fusion protein and TNS4. (C) Proximity ligation assay (PLA) in pancreatic cancer cells showing punctate GMFG-TNS4 proximity signal. (D) Confocal immunofluorescence after incubation with red-labeled recombinant GMFG and subsequent TNS4 staining. Scale bars is 20μm.


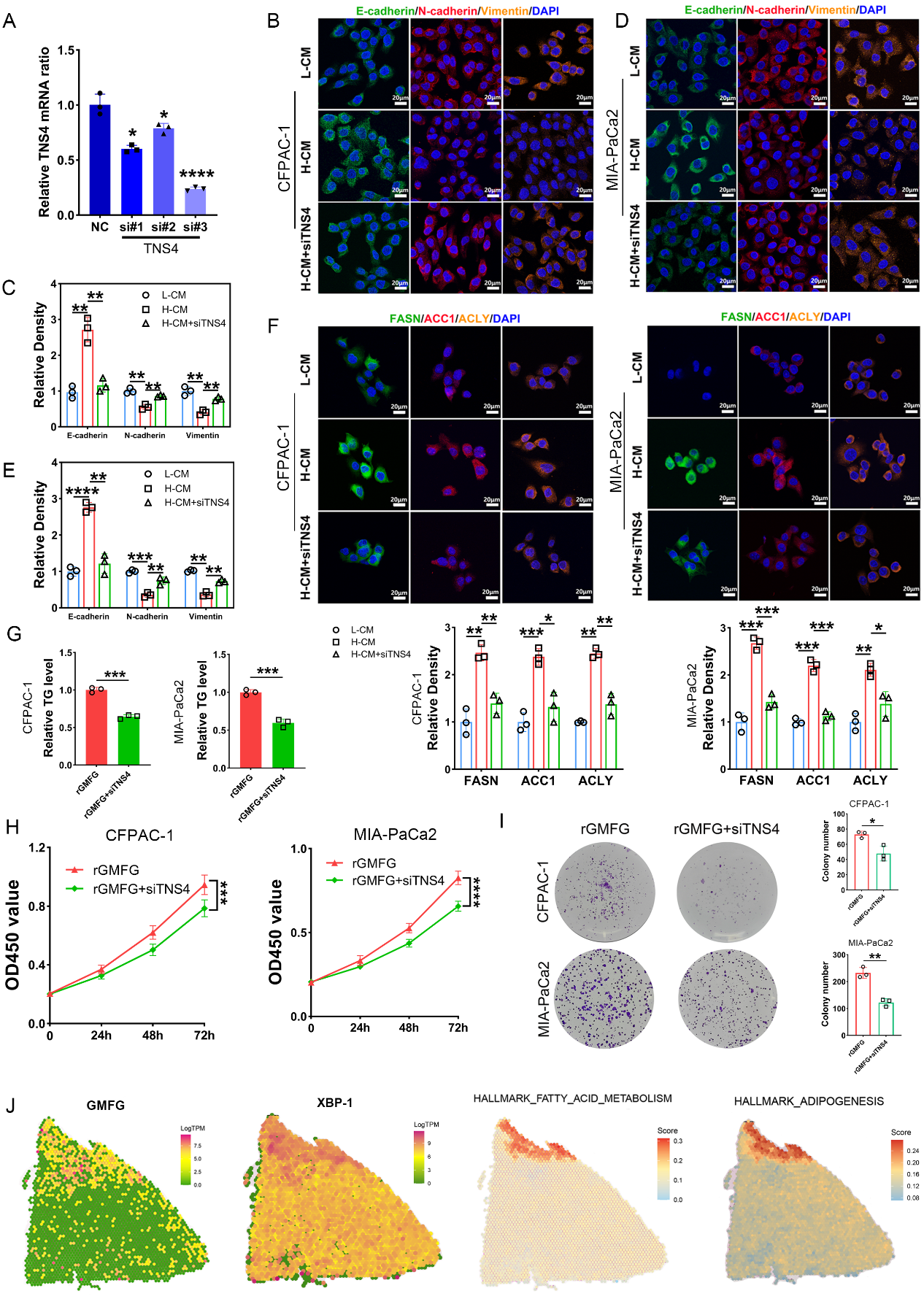


**Figure. S12. TNS4 depletion reverses GMFG-associated epithelial/lipogenic programs and spatially relates to fatty-acid metabolism in liver metastasis.** (A) Validation of human TNS4 knockdown efficiency in CFPAC-1 cells by siRNA (n=3). (B–F) Immunofluorescence analyses showing altered epithelial/mesenchymal marker expression after TNS4 knockdown (n=5). (G) Triglyceride quantification after TNS4 knockdown (n=3). (H, I) CCK-8 (n=8) and colony formation (n=3) assays showing reduced proliferation after TNS4 knockdown. (J) Spatial transcriptomics dataset (GSE281288) showing spatial localization of GMFG, XBP1, and fatty acid metabolic/lipogenic processes in pancreatic cancer liver metastases. Scale bars are indicated in the images. All data are presented as mean ± SD. Statistical analysis was performed using two-tailed Student’s t-test (two groups) or one-way ANOVA. *P<0.05, **P<0.01, ***P<0.001, ****P<0.0001.


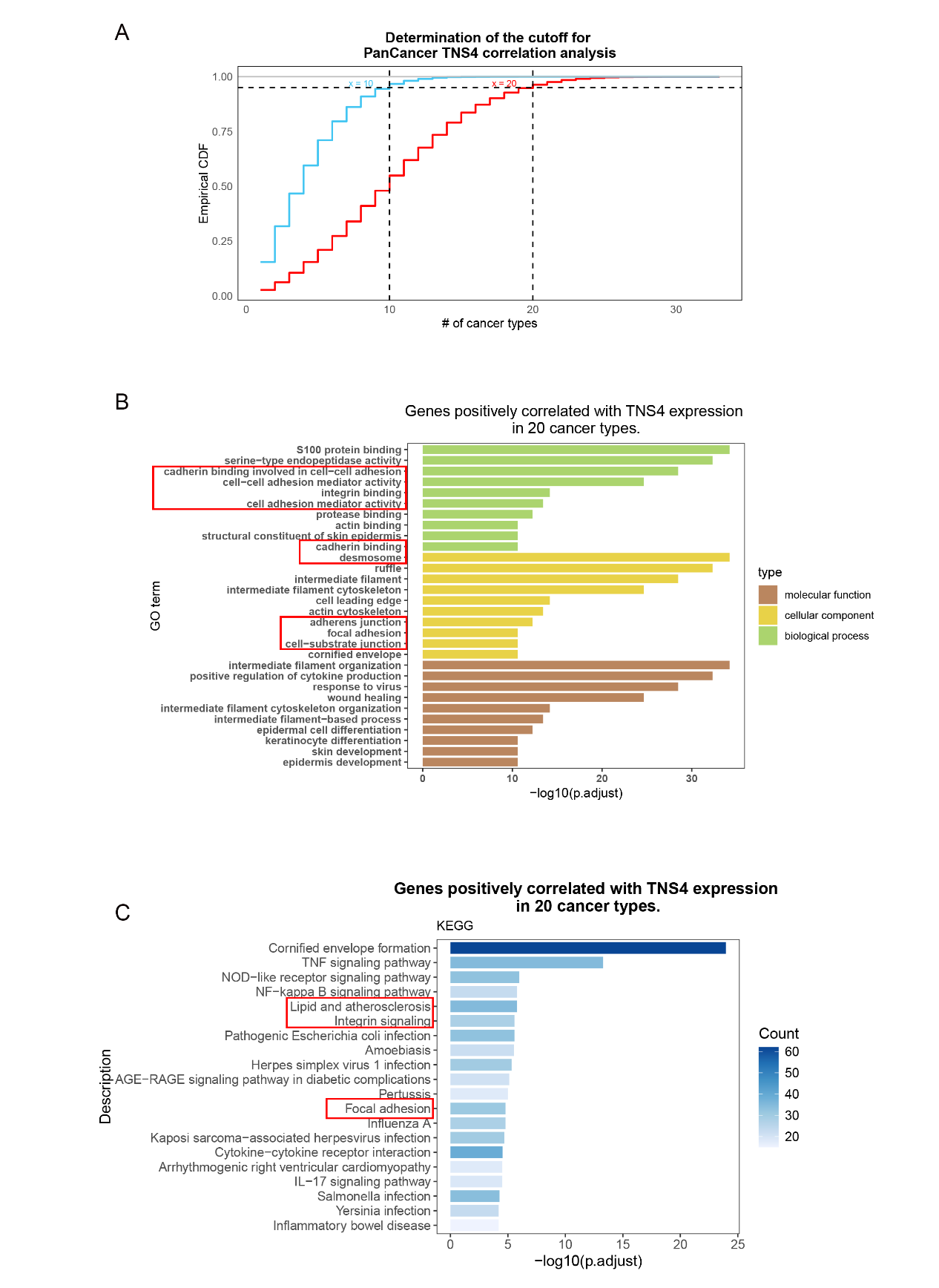


**Figure. S13. Pan-cancer correlation analysis links TNS4 to focal adhesion–related programs.** (A) Empirical cumulative distribution function (ECDF) plot used to determine the cutoff for pan-cancer TNS4 correlation analysis; dashed vertical lines indicate representative thresholds (e.g., correlation observed in ≥10 or ≥20 cancer types) used to define a robust TNS4-associated gene set. (B) Gene Ontology (GO) enrichment of genes positively correlated with TNS4 expression across 20 cancer types, highlighting terms associated with cell–cell/cell–substrate adhesion, cadherin/integrin binding, actin cytoskeleton organization, and focal adhesion. (C) KEGG pathway enrichment of the same TNS4-positively correlated gene set, showing significant enrichment in focal adhesion and integrin signaling pathways (along with other cancer-associated inflammatory pathways).


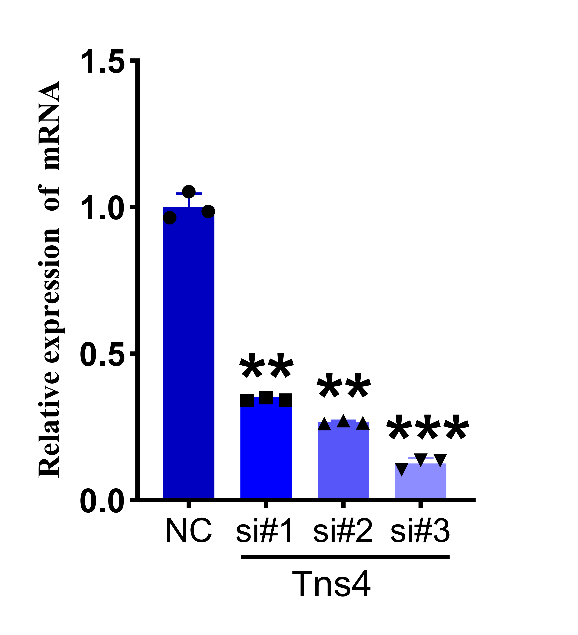


**Figure. S14. qPCR validation of mouse Tns4 knockdown efficiency.** Quantitative PCR analysis confirming the knockdown efficiency of mouse Tns4 siRNA in indicated conditions (n=3). All data are presented as mean ± SD. Statistical analysis was performed using two-tailed Student’s *t*-test (two groups) or one-way ANOVA. ***P*<0.01, ****P*<0.001.


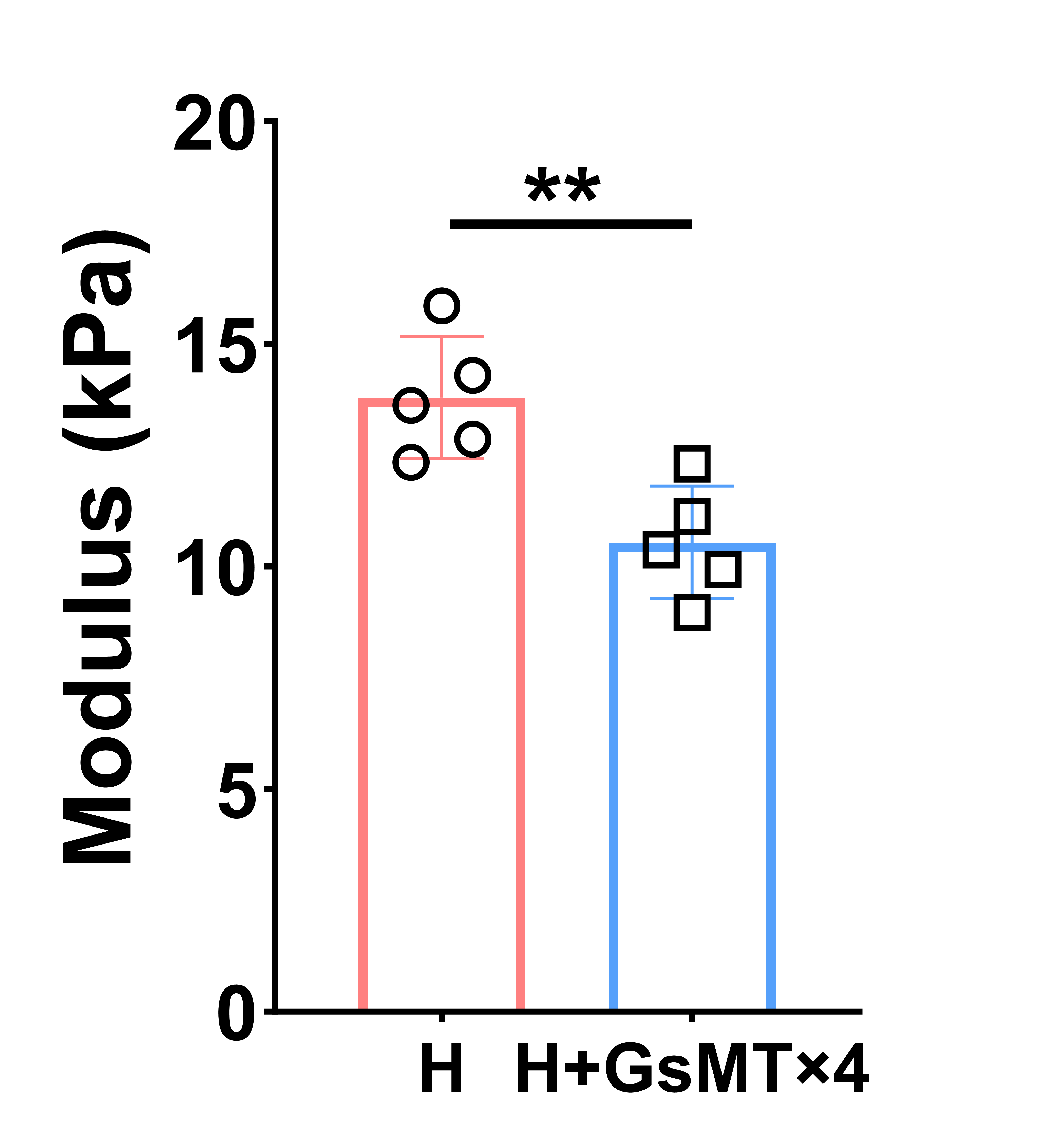


**Figure. S15. GsMTx4 treatment partially reduces overall liver stiffness in mice with stiff livers.** Quantification of overall liver tissue stiffness measured by unconfined compression testing in the H and H+GsMTx4 groups. Data are presented as mean ± SD. Statistical analysis was performed using two-tailed Student’s t-test. **P<0.01.


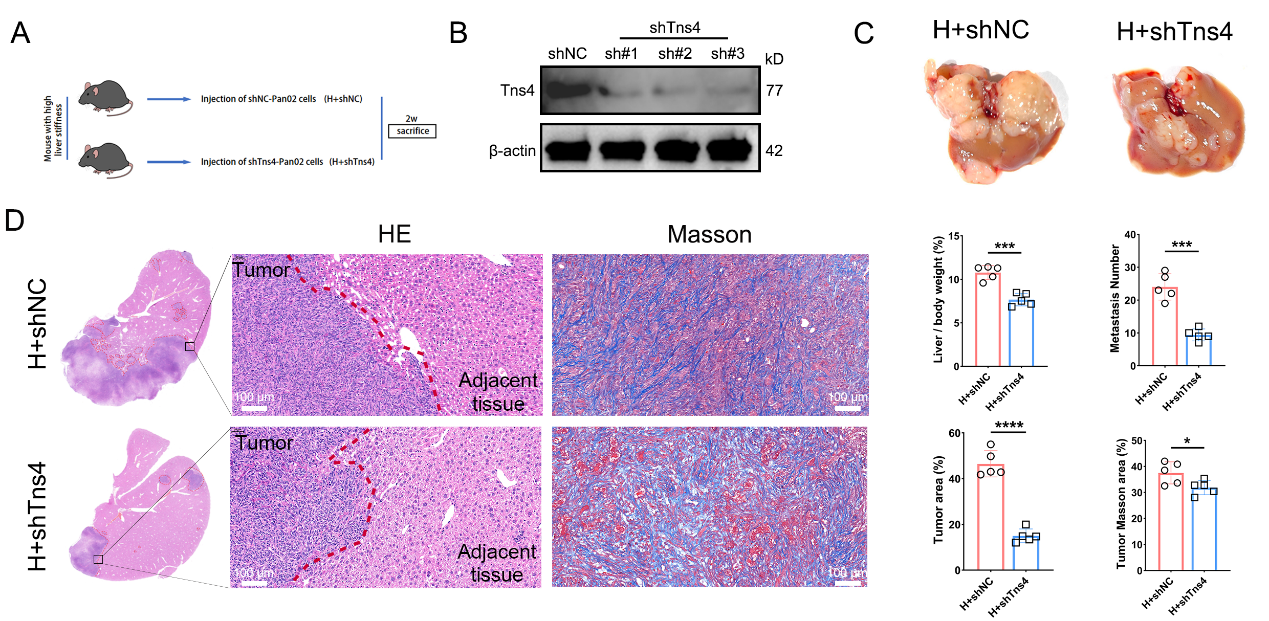


**Figure. S16. Stable knockdown of Tns4 in Pan02 cells suppresses liver metastatic burden in mice with stiff livers.** (A) Schematic illustration of the in vivo experimental design. Pan02 cells stably expressing shNC or shTns4 were injected into mice with pre-established high liver stiffness, and mice were sacrificed at 2 weeks. (B) Western blot validation of Tns4 knockdown efficiency in Pan02 cells stably transduced with shTns4 lentiviral constructs. β-actin was used as the loading control. (C) Representative gross liver images. (D) Representative H&E and Masson’s trichrome staining of metastatic lesions from the two groups and quantification of liver/body weight ratio, metastatic nodule number, collagen deposition (tumor Masson area) and tumor area in the H+shNC and H+shTns4 groups. Scale bars are indicated in the images. All data are presented as mean ± SD. Statistical analysis was performed using two-tailed Student’s t-test. *P<0.05, ***P<0.001, ****P<0.0001.

Table S1. Available clinicopathological information for the representative clinical case shown in Fig. 1A.

| **Sex** | Male | **Metastatic lesion** | Circular,  diameter~1cm |
| --- | --- | --- | --- |
| **Age** | 74 | **Liver background** | Localized stiffening,  no formal grade |
| **Primary tumor** | head and neck, 38*36mm | **presence of HBV infection** | No |

Table S2. List of primary antibodies used in the current study.

| **Primary antibody** | **Company** | **Cat. NO.** | **Application** |
| --- | --- | --- | --- |
| α-SMA | Proteintech | 14395-1-AP | IHC/IF/WB |
| COL1 | Proteintech | 14695-1-AP | IHC/WB |
| Ki67 | Proteintech | 27309-1-AP | IHC |
| Piezo1 | Proteintech | 15939-1-AP | IF/WB |
| GAPDH | Proteintech | 10494-1-AP | WB |
| β-actin | Proteintech | 66009-1-Ig | WB |
| E-cadherin | Proteintech | 20874-1-AP | IHC/IF/WB |
| N-cadherin | Proteintech | 22018-1-AP | IHC/IF/WB |
| Vimentin | Proteintech | 10366-1-AP | IHC/IF/WB |
| FASN | Proteintech | 10624-2-AP | IHC/IF/WB |
| ACC1 | Proteintech | 21923-1-AP | IHC/IF/WB |
| ACLY | Proteintech | 67166-1-Ig | IHC/IF/WB |
| IRE1α | Zenbio | 680345 | WB |
| p-IRE1α | Zenbio | R26310 | WB |
| XBP-1 | Proteintech | 24168-1-AP | WB |
| CHOP | Proteintech | 15204-1-AP | IF/WB |
| GRP78 | Proteintech | 11587-1-AP | IF/WB |
| GMFG | Proteintech | 13625-1-AP | IHC/WB |
| TNS4 | Proteintech | 11580-1-AP | IHC/WB |
| FAK | Abcam | Ab40794 | WB |
| p-FAK | Abcam | Ab81298 | WB |
| AKT | Proteintech | 10176-2-AP | WB |
| p-AKT | Proteintech | 66444-1-Ig | WB |
| Calnexin | Proteintech | 10427-2-AP | WB |
| CD9 | Proteintech | 20597-1-AP | WB |
| CD63 | Proteintech | 25682-1-AP | WB |

Table S3. List of primer sequence.

| **Oligo Name** | **Sequence (5’ to 3’)** |
| --- | --- |
| Piezo1-F | GAG CTG GTG AAG GTG AAG AA |
| Piezo1-R | GGC AAA GTC CTC GAA GAT GA |
| COL1A1-F | AAA GAT GGA CTC AAC GGT CTC |
| COL1A1-R | CAT CGT GAG CCT TCT CTT GAG |
| COL1A2-F | CTC CAT GGT GAG TTT GGT CTC |
| COL1A2-R | CTT CCA ATA GG ACCA GTA GGA C |
| LOXL2-F | CTG GGA GAA CCT GGA GAA GA |
| LOXL2-R | GCT GGT TCT GGT GAT CTT CC |
| CDH1-F | TCT GCT GCT CTT GCT GTT TCT TC |
| CDH1-R | TCT CCG CCT CCT TCT TCA TCA TAG |
| CDH2-F | CAG TCC AAG TCC GAG TCA AG |
| CDH2-R | TGT TCA GGA TCA CCT TCG TC |
| FASN-F | CCA TCT ACA ACA TCG ACA CCA G |
| FASN-R | CTT CCA CAC TAT GCT CAG GTA G |
| ACACA-F | TGC CTC CAC ACT CAC TCT TCC |
| ACACA-R | ACC TGC TGC CTG TCT ACA CTC |
| ACLY-F | CAG AAT CGG TTC AAG TAT GCT C |
| ACLY-R | AAG TTT TCC ACG ACG TTT GAT C |
| IREα-F | CGT GAG CGA CAG AAT AGA AAA G |
| IREα-R | GCT TCT TAT TTC TCA TGG CTC G |
| XBP-1-F | CTT GTA GTT GAG AAC CAG GAG T |
| XBP-1-R | CCC AAC AGG ATA TCA GAC TCT G |
| CHOP-F | GAG AAT GAA AGG AAA GTG GCA C |
| CHOP-R | ATT CAC CAT TCG GTC AAT CAG A |
| GRP78-F | CAG TTG TTA CTG TAC CAG CCT A |
| GRP78-R | CAT TTA GGC CAG CAA TAG TTC C |
| ATF4-F | ATG GAT TTG AAG GAG TTC GAC T |
| ATF4-R | AGA GAT CAC AAGT GT CAT CCA A |
| ATF6-F | CTG ATGG CT GTT CAA TAC ACA G |
| ATF6-R | GAT CCC TTC GAA ATG ACA CAA C |
| GAPDH-F | CAG GAG GCA TTG CTG ATG AT |
| GAPDH-R | GAA GGC TGG GGC TCA TTT |

Table S4. List of siRNA sequence

| Name | Sequence (5’-3’) | |
| --- | --- | --- |
| siPiezo1#1 | sense | CCAAGUACUGGAUCUAUGU |
|  | antisense | ACAUAGAUCCAGUACUUGG |
| siPiezo1#2 | sense | CUCAAGUACUUCAUCAACU |
|  | antisense | AGUUGAUGAAGUACUUGAG |
| siPiezo1#3 | sense | CCAAGAAGUACAAUCAUCU |
|  | antisense | AGAUGAUUGUACUUCUUGG |
| siGMFG#1 | sense | GCUCACAAAGGUGUUCGAA |
|  | antisense | UUCGAACACCUUUGUGAGC |
| siGMFG#2 | sense | GGGACUGAAUUCCUGAUGU |
|  | antisense | ACAUCAGGAAUUCAGUCCC |
| siGMFG#3 | sense | GGUUCGUGGUUUACAGCUA |
|  | antisense | UAGCUGUAAACCACGAACC |
| siTNS4#1 | sense | GGACAGCUCUUCAUACCGA |
|  | antisense | UCGGUAUGAAGAGCUGUCC |
| siTNS4#2 | sense | CAGAGGACCUUGACUCCUA |
|  | antisense | UAGGAGUCAAGGUCCUCUG |
| siTNS4#3 | sense | GCCAUCAGUCAUCUUCCAGAU |
|  | antisense | AUCUGGAAGAUGACUGAUGGC |
| siTns4#1 | sense | GCUGCAUGUACGUCAAGUA |
|  | antisense | UACUUGACGUACAUGCUGC |
| siTns4#2 | sense | GGAGACAGUGGUGUCUUUA |
|  | antisense | UAAAGACACCACUGUCUCC |
| siTns4#3 | sense | CCUCAUGUUAUCUGGGAACUU |
|  | antisense | AAGUUCCCAGAUAACAUGAGG |
